# Carbon cycle perturbations and environmental change of the middle permian and Late Triassic Paleo-Antarctic circle

**DOI:** 10.1038/s41598-024-60088-5

**Published:** 2024-04-28

**Authors:** Wahyuningrum Lestari, Aisha Al-Suwaidi, Calum P. Fox, Vivi Vajda, Dominik Hennhoefer

**Affiliations:** 1 Japan Agency for Marine-Earth Science and Technology (JAMSTEC), 2-15 Natsushimacho, Yokosuka, Kanagawa 237-0061 Japan; 2 Department of Paleobiology, Swedish Museum of Natural History, Svante Arrhenius V. 9, Stockholm, Sweden; 3 Hessisches Landesmuseum Darmstadt, Friedensplatz 1, 64283 Darmstadt, Germany; 4 Present Address: Department of Earth Sciences, Khalifa University of Science and Technology, Abu Dhabi, UAE

**Keywords:** Carnian Pluvial Episode, Carbon isotopes, Southern Hemisphere, Middle Permian, Norian, Carbon cycle perturbation, Environmental perturbation, Carbon cycle, Palaeoclimate, Geochemistry

## Abstract

During the middle Permian through the Triassic, Tasmania moved from paleo-latitudes of 78° to 69°S, wedged between Antarctica and Australia, within the paleo-South polar circle. During this time, significant global carbon cycle disturbances triggered major environmental and climatic changes and mass extinction events globally. The Bicheno-5 core from Eastern Tasmania, Australia, provides the opportunity to examine middle Permian and Upper Triassic sediments from the paleo-Antarctic, using high-resolution organic carbon isotope (δ^13^C_TOC_) chemostratigraphy, pXRF, and sedimentology, combined with new palynological data integrated with the existing radiometric age model. While there is a significant unconformity in the Upper Permian to the middle Triassic associated with eustatic sea-level fall as a result of regional uplift in eastern Australia, three distinct carbon isotope excursions (CIEs), characterized by negative shifts of up to − 6 ‰ were identified; the middle Permian Guadalupian Carbon Isotope Excursions (G-CIE), the Carnian Pluvial Episode (CPE), and the mid-Norian Event (MNE). These three events highlight a significant climate shift through glacial and interglacial cycles to warmer non-glacial intervals in the Late Triassic, with evidence of the polar record of the Carnian Pluvial Episode and the mid-Norian Event, which are poorly studied in the Southern Hemisphere, specifically within the Paleo-Antarctic circle.

The end-Permian Mass Extinction (EPME, 252.1 Ma^1^) represents an interval of significant disruption to ecosystems and carbon cycle driven by the emplacement of the Siberian Traps Large Igneous Province (LIP; eg.^2,3^) and also influenced by other coeval magmatic events^4,5^) marked by an estimated extinction of 97% of marine organisms and on land 49% of tetrapod families on land^6–10^ Following the EPME was a period of recovery that is thought to have lasted around 5 million years, marked by more arid conditions globally and with notable recovery by the middle Triassic (cf.^7,9,11^). While the EPME is considered one of the most significant extinction events on Earth, before the onset of the event and following the prolonged recovery, the Earth experienced other major carbon cycle perturbations associated with substantial environmental and climatic change, including, but not limited to, Olsen’s Extinction (~ 273 Ma late Cisuralian to early Guadalupian^12,13^), the Guadalupian Carbon Isotope Excursion (G-CIE) ending in the Capitanian Extinction Event (~ 262 Ma^9,14–23^), the Carnian Pluvial Episode (CPE, ~ 232–234 Ma^24–36^) and the mid-Norian Event (MNE, ~ 215 Ma^37,38^) leading up to the end-Triassic Mass Extinction (eg.^39–42^). Records of these events are relatively common from the Northern Hemisphere (eg.^27,43–50^ ) but there is a lack of high-resolution records of these events from high-latitude Southern Hemisphere localities. Here we present new evidence of these events from a drill core recovered in Eastern Tasmania, known as Bicheno-5, including high-resolution carbon isotope chemostratigraphy, elemental and sedimentological data (pXRF and hylogging), as well as a palynological assessment. These data are combined with previously published geochronology, allowing the identification of three major carbon cycle and environmental perturbations; the middle Permian G-CIE, the CPE, and the MNE. Importantly these records highlight the impact of carbon cycle perturbations in the Paleo-Antarctic circle, as, during the middle Permian, Tasmania was at a paleo-latitudes of 78°S, moving Northward to 69°S by the Late Triassic (Fig. 1). (Supplementary file 2)

**Figure 1:**
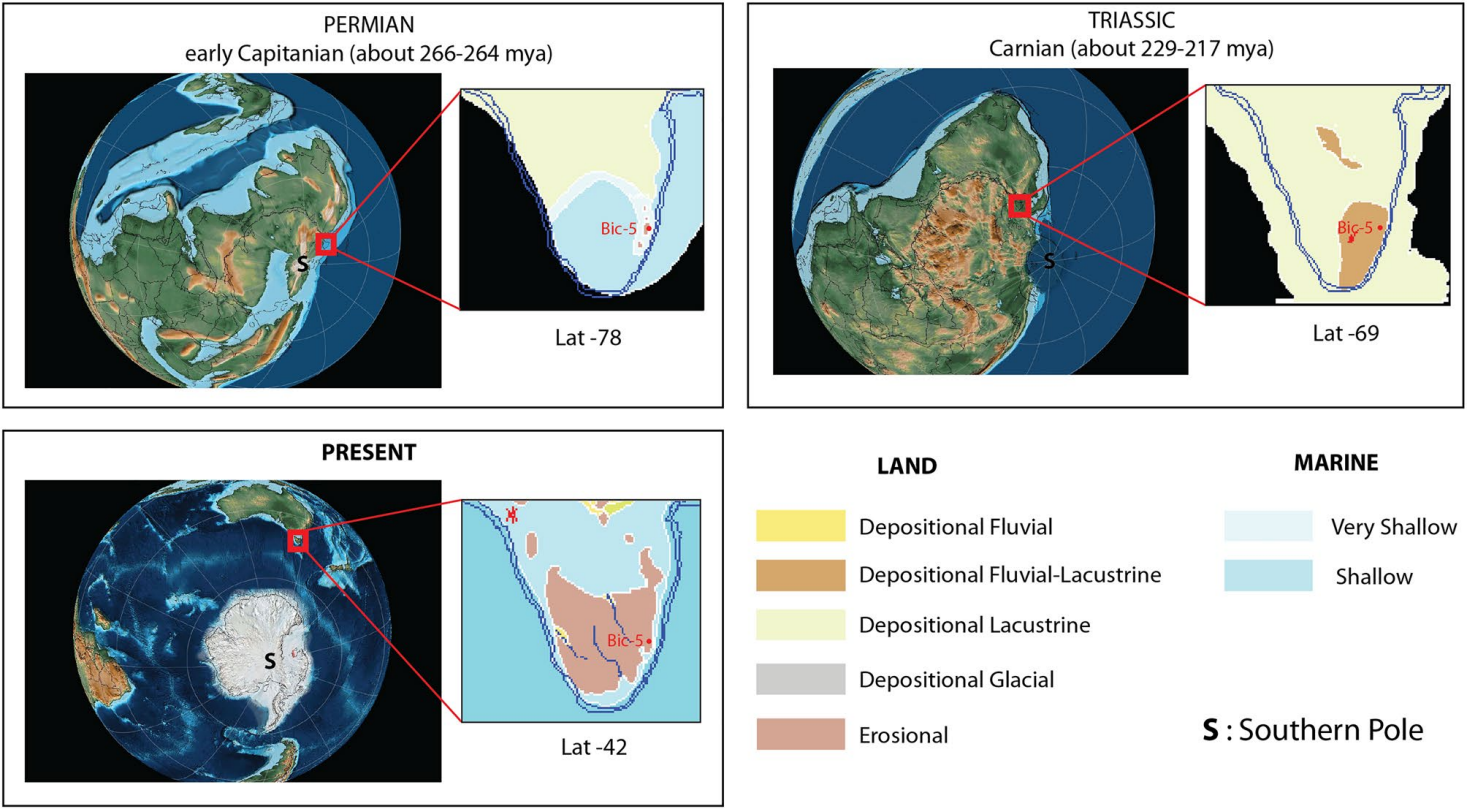
Paleo-position and paleoenvironment of Tasmania for the middle Permian (early Capitanian, 266–264 ma), late mid-Triassic (Carnian, 229–217 Ma), and present with regards to Bicheno-5 core position. Paleogeographic figures were generated using GPlates^98^ using global paleogeoghraphic map reconstructions of^99,100^ .

The Guadalupian (ca. ~ 259.8 Ma^51^) mass extinction event is associated with a significant negative CIE of > − 5 ‰ disrupting the otherwise positive carbon isotope trend in mid-Permian sediments (cf.^13–18,21,49,52,53^). This event is typically interpreted to be associated with an overall climatic cooling, a drop in biological productivity both on land and in the oceans, and the demise of many photosynthetic marine taxa^14,16,21,22,51,52^, formation of ocean stratification and anoxia^20,54^, sea-level change^14,17,23^, and methane release^48,55^. The Guadalupian CIE (G-CIE) coincides with the emplacement of the Emeishan LIP in Southwest China between ~ 257–260 Ma (eg.^18,51,53,56–58^). However, the link between the emplacement of the Emeishan LIP, the G-CIE, extinction, and evidence of the global extent of the event is still heavily debated, owing to the lack of chronologically well-constrained high-resolution carbon isotope records.

The CPE, (ca. ~ 232–234 Ma^59^) is a defining point in the Late Triassic, marked by a significant climate shift to more wet and humid conditions globally. It is associated with carbon cycle perturbations of up to − 6 ‰ (cf.^24–26,31,33–36,60^), changes in land biota including a notable global shift from a conifer-dominated forest community to a fern-dominated community suggestive of increased humidity^27^, and the extinction of some Triassic crinoid taxa^61^. The CPE is also considered a critical event leading to the rise and diversification of dinosaurs^59,62^. The rapid switch between arid to more humid conditions, caused significant plant extinctions and subsequent ecological shifts, enabling enhanced diversification, and radiation, especially of herbivorous dinosaurs^63,64^. It is linked to the emplacement of the Wrangellian Terrian LIP in the North Pacific (cf.^24,65^), however, more detailed studies are needed to understand the global nature and impact of the CPE. To date carbon isotope records of the CPE are limited to China (eg.^31,34–36,65^), Japan^26^, the UK^33,66^, the Barents Sea^30^, the NW Tethys (Italy and Hungary^24,25,60^), the Indian Himalayas^46^, and Argentina^50^. The evidence of the CPE from Argentina derives from a relatively low-resolution carbonate carbon-isotope record and does not show the typical CPE stepped excursion; however, it does provide a relatively precise U–Pb age of 234.47 ± 0.44 Ma (Carnian) within the lower Los Rastros Formation, above which evidence for the CPE is recorded. This section also provides evidence of the hydrological cycle intensification based on clay mineralogy and paleontological evidence of the rapid diversification of dinosaurs during the Carnian^50^.

The MNE carbon cycle perturbation typically has a magnitude of − 2–3 ‰ (Onoue et al., 2016) and evidence of a drop in atmospheric CO_2_ during a period typically marked by relatively high atmospheric CO_2_ values, as estimated from pedogenic carbonate data^67^. The climate was mainly dry, with a gradual increase in humidity beginning in the late Norian^68–70^. An extraterrestrial bolide impact at ~ 215.5 Ma identified from the 90-km-diameter Manicouagan crater in Canada is postulated as the main driver of this event (eg.^37,71–73^). The mid-Norian event is associated with intense aridity in the tropical desert belts; unstable climate^67^; a shutdown of primary productivity in the oceans, following the impact, as demonstrated by the decline in biogenic silica and burial fluxes of radiolarian silica, and the proliferation of siliceous sponges^73^. The shift to more humid climatic belts, which starts in the Norian, is often associated with the dispersal of dinosaurs further north^67^ and ultimately ended the prolonged arid climate that dominated the Triassic.

These three events are characterized by significant ecological and environmental perturbations. In order to understand how these events affected global processes such as weathering rates, the carbon cycle, and climate, we need to examine records globally. Here we present a new high-resolution record from the Bicheno-5 core, Llandaff coalfield, Eastern Tasmania, Australia (Fig. 1) representing a unique view into the southern polar circle during these critical intervals of Earth’s History.

## Result age, lithostratigraphy, and geochemistry of Bicheno-5

Bicheno-5 comprises 300 m sediments belonging to the Lower and Upper Parmeener Supergroup spanning the Guadalupian (middle Permian) to the Norian (Late Triassic), with a significant unconformity spanning the latest middle Permian to late Ladinian^74^. The sediments were deposited in the intracratonic Tasman Basin, located in the most distal part of a foreland basin system on the convergent Panthalassan Margin that stretched along Gondwana from western Argentina to northern Queensland^75^(Fig. 1).

**Figure 2:**
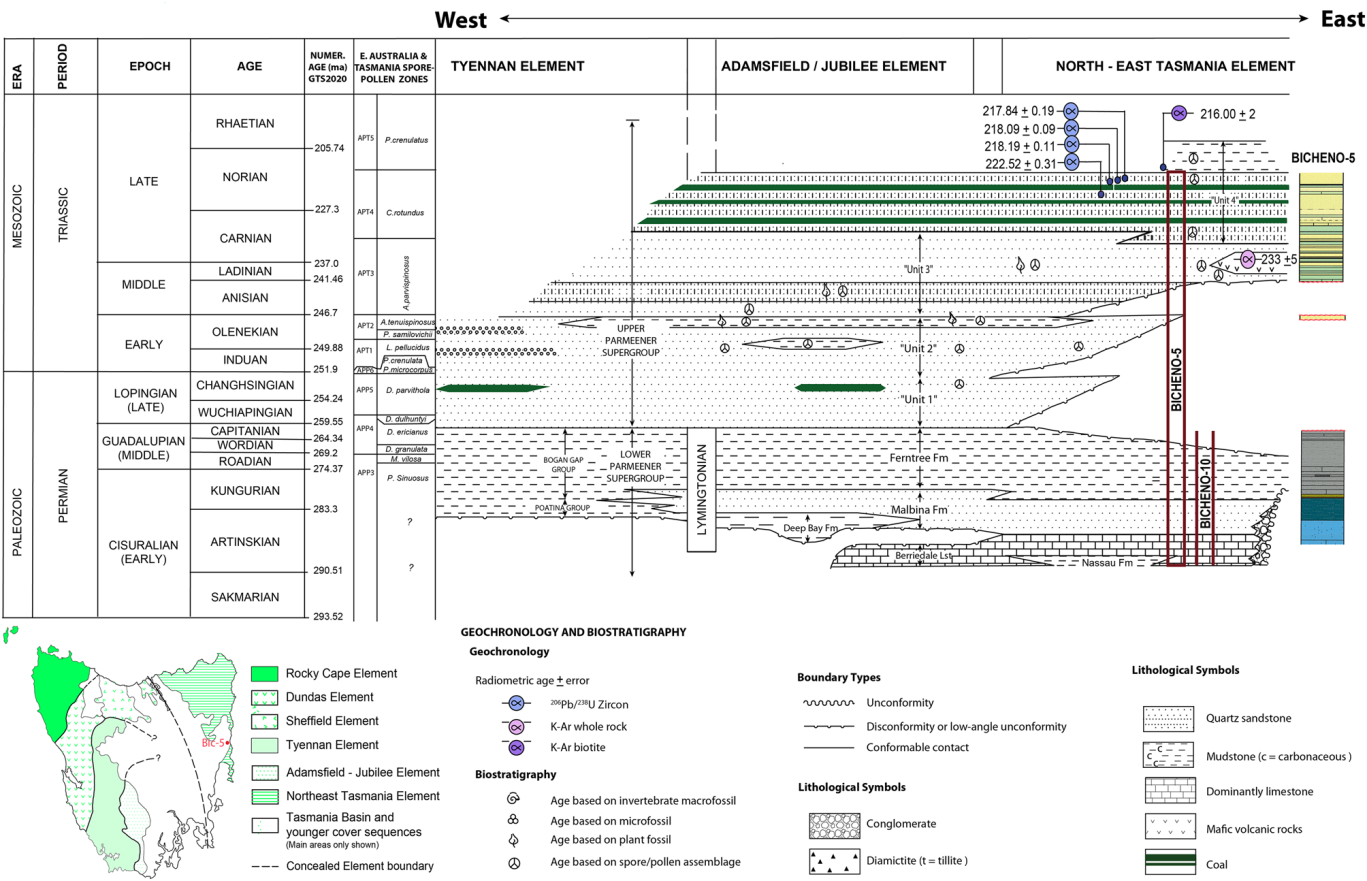
General Permian and Triassic stratigraphy of the Tasmania Basin (modified from^101^ from www.mrt.tas.gov.au, State of Tasmania). The uppermost-300-m of the Bicheno-5 core consists of an Upper Freshwater Sequence belonging to the Upper Parmeener Supergroup and an Upper Marine Sequence belonging to the Lower Parmeener Supergroup. The guide rectangle in the North-East-Tasmania Element indicates the approximate position of the Bicheno-5 core. The lithostratigraphic image of the core indicates the location of significant unconformities in the lower part of the core. Geochronology follows^77^. Dates of boundaries are based on^102^. Australian Permian palynostratigraphic zonation is based on^81–83^.

The upper part of the core (Unit 4, Fig. 2) contains a welded tuff below Jurassic dolerites that has been dated to 214 ± 1 Ma using K-Ar^76^recalculated to 216 ± 2 Ma^77^ (Fig. 2). More recent work by^77^ of a tuff stratigraphically lower than the level dated by^76^ in correlative cores GY27, FT28B, and in outcrop successions in the Dennison River area has provided new U–Pb ages of 217.84 ± 0.19 Ma, 218.19 ± 0.11 Ma, and 218.09 ± 0.09 Ma. A U–Pb date of 222.52 ± 0.31 Ma is also provided from a horizon that is stratigraphically lower than the Denison Rivulet tuff and the 233 ± 5 Ma K–Ar date determined from the basalt within Unit 3^78^ .The relative position of the ages within the Upper Parmeener Supergroup and correlation to Bicheno-5 is shown in Fig. 2 and Figure SI–S2. Similarly, the Mount Nicholas Coal Measure in N-E Tasmania shows complementary palynological evidence^77^ to that presented below for Bicheno-5, confirming the Late Triassic, Carnian–Norian age of the upper part of the core.

The middle Permian sediments include the marine and glaciomarine strata known as the upper marine sequence of the Lower Parmeener Supergroup^79^ (Figs. 2,3 and 4, 217–300 m). While the Late Triassic sediments represent the fluvial deposits of the upper freshwater sequence of the Upper Parmeener Supergroup^79^ (Fig. 2, 6.5–217 m, Fig. 3). In some parts of North-eastern Tasmania, there is a hiatus in the Upper Permian to Lower Triassic sedimentary successions, representing a break in sedimentation possibly due to eustatic sea-level fall as a result of regional uplift in eastern Australia^74^ or post-depositional erosion within the lower section (Unit 1) of the Upper Freshwater Sequence. Thus the record of the End Permian Extinction and the Permian–Triassic boundary is missing in Bicheno 5.

**Figure 3:**
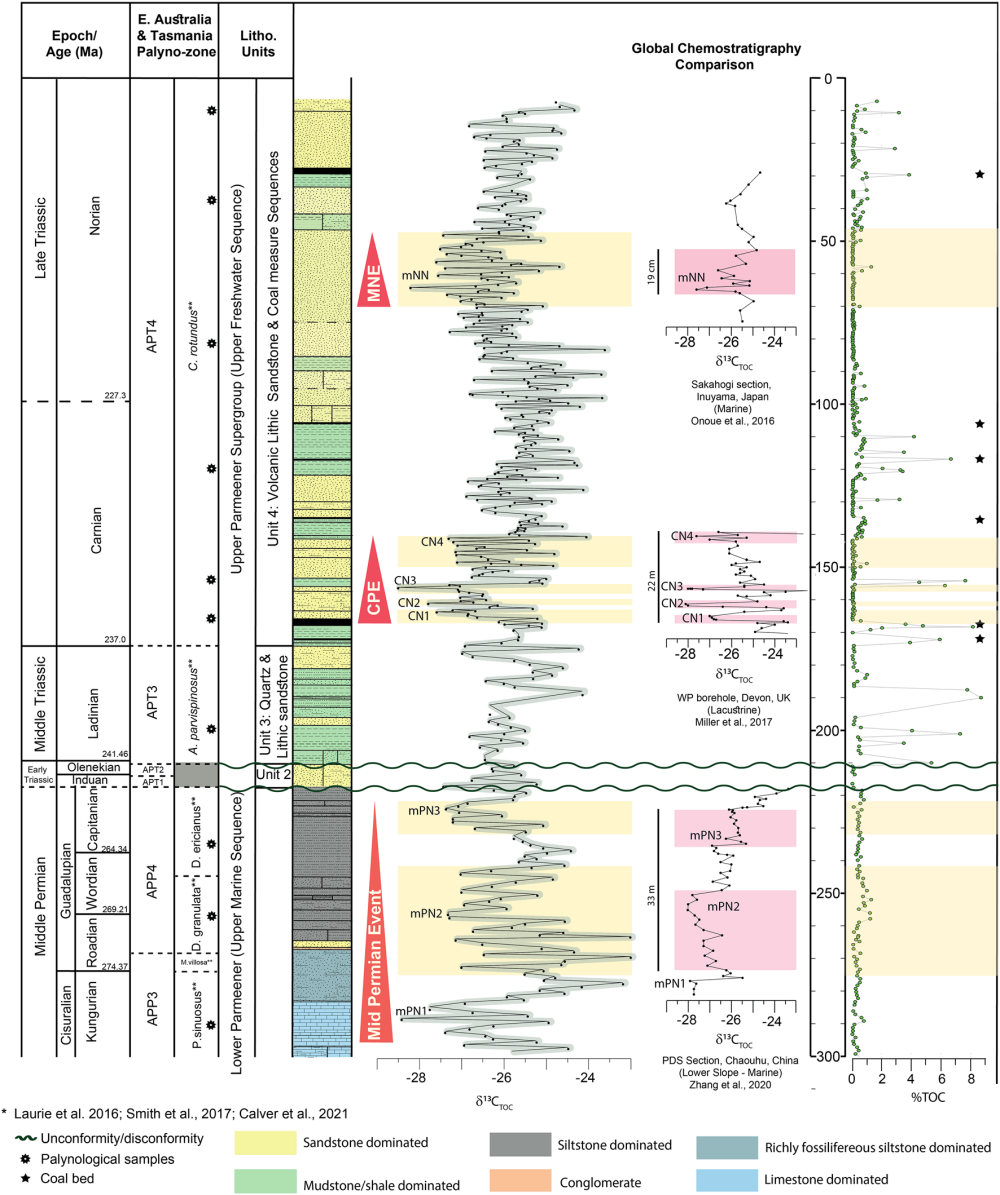
Schematic illustration of the Bicheno-5 core through middle Permian to Late Triassic. Figures show graphical log, carbon isotope data, and Total Organic Carbon (TOC). (**a**) Chemostratigraphic comparison of the mid-Permian event to data from^13^ (**b**) The Carnian Pluvial Episode (CPE), comparison with^33^, and (**c**) Mid-Norian Event (MNE) comparison with^37^ (**d**) palynological zonation based on the palynological assemblages herein. Abbreviations are outlined in the text.

**Figure 4:**
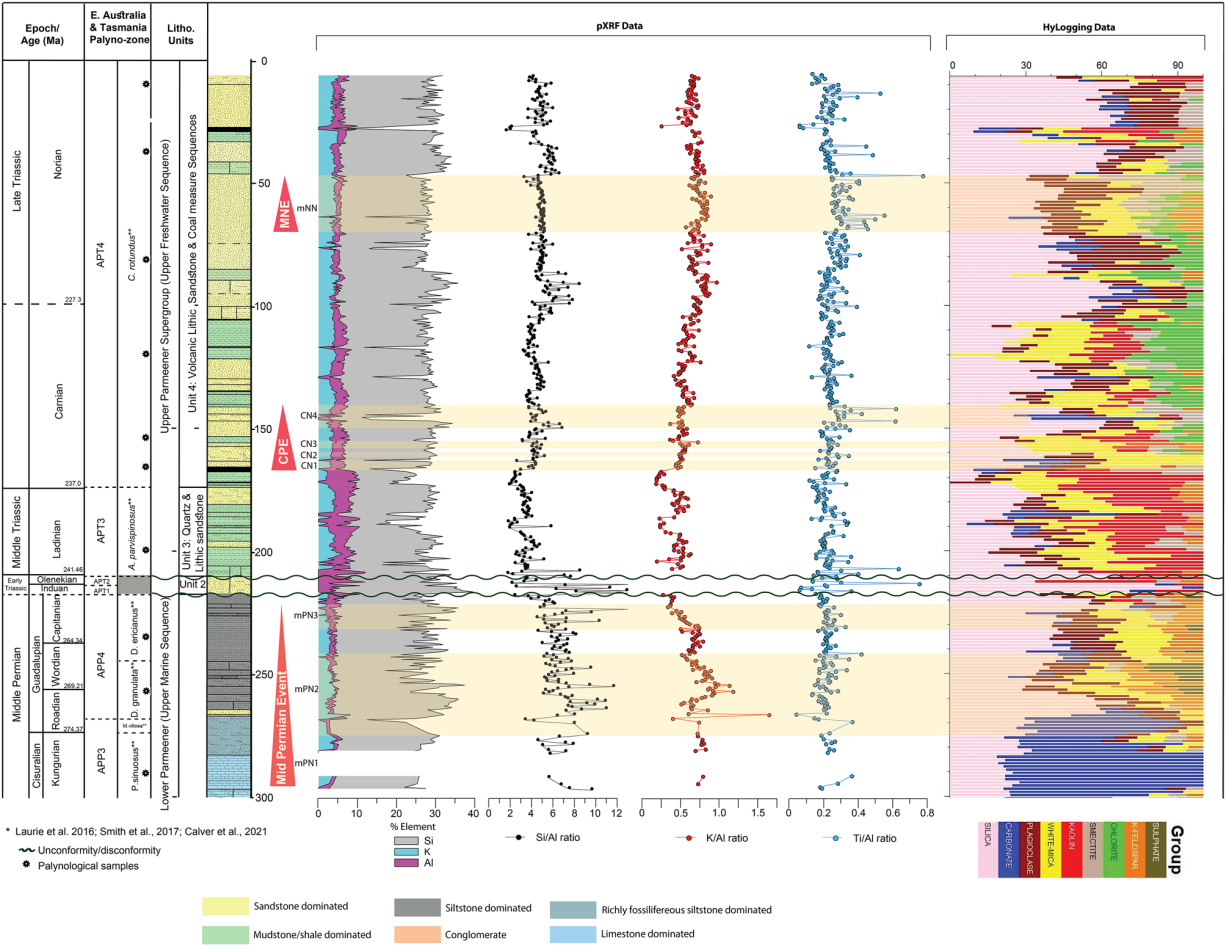
Schematic illustration of the Bicheno-5 core through middle Permian to Late Triassic. Figures show graphical log, Si, Al, and K concentrations, Si/Al, K/Al, and Ti/Al ratios in regards to: (**a**) mid-Permian event, (**b**) Carnian Pluvial Episode (CPE), and (**c**) Mid-Norian Event (MNE). Palynological zonation based on the palynological assemblages herein. Abbreviations are outlined in the text.

The middle Permian of N-E Tasmania (Kungurian–Capitanian; interval 217–300 m).

The Lower Parmeener Supergroup in Bicheno-5 consists primarily of the upper marine sequence, which includes calcarenite limestone (282.1–300 m), richly-fossiliferous siltstone (264.5–282.1 m), conglomerate followed by glauconitic sandstone occurring at 267.1–264.3 m, and siltstone-dominated deposit with some evidence of dropstones (217–264.3 m). Palynological data from the Upper Marine Sequence of Bicheno-5 and the nearby correlative Bicheno-10 core^80^ (Fig. 2; Fig. SI–S3), indicate a Cisuralian, specifically Kungurian to Guadalupian age (~ 283.3 to 259.55 Ma, Australian Palynostratographic Permian zone, APP 3 and 4^80^ with updates adapted from ^81–83^. These palynostratigraphic stages have in recent years also been correlated to high precision U–Pb geochronology across the Sydney Basin, further constraining and confirming the assignment of ages of these zones with the Geological Time Scale (cf.^82,83^)).

Three samples were analyzed for palynology from the interval of 289.1 m, 257.8 m, and 233.3 m. The sample from 233.3 m displays a well-preserved and abundant palynoflora of low diversity, overwhelmingly dominated by terrestrial palynomorphs and with only a few marine/brackish water algal taxa. Small, non-taeniate bisaccate pollen of possible Voltsialean affinity dominate the assemblage but monosaccate pollen also occur. Trilete spores are subordinate, but importantly the zonal key taxon for the top of APP4 *Didecitriletes ericianus* is present, indicating a Capitanian age, and so are smooth, trilete spores, including *Microbaculatispora* spp. Other genera of importance are *Vittatina* and several specimens of monosulcate pollen, possibly representing pteridospermales (‘seed-ferns’).

In both samples, 289.1 m and 257.8 m, the assemblage is poorly preserved with a high portion of amorphous organic matter due to post-depositional degradation. Specifically, in the sample from 257.8 m, the assemblage is extremely sparse, although several specimens of the key-taxon *Phaselisporites cicatricosus* were identified. This spore has a first appearance datum at the base of Zone APP3 (Fig. 2).

The oldest sample, 289.1 m, potentially from the *P. sinuosus* assemblage zone, hosts a palynological assemblage typical of a marine depositional environment. Structured palynomorphs, including the algae *Tasmanites* spp., and non-taeniate bisacate pollen grains occur together with coniferous phytoclasts. However, no zonal key taxa were identified.

Total Organic Carbon (TOC) is relatively low, < 1%, in the Lower Parmeener Supergroup, especially in the limestone-dominated portion of the Permian (268–300 m; Fig. 3). However, the TOC increases slightly to ~ 1.3% in some of the siltstone deposits. The C/N ratio is mostly below 20, with only three points above this value at depths of 244.35 m, 289.18 m, and 297.2 m, measuring 21.47%, 32.5%, and 35.7%, respectively (Fig. SI–S4). The K/Al ratio shows a two-stage decrease at 242–258 m and 223–230 m, whereas Si/Al and Ti/Al ratio are more stable with average values of 7 and 0.3, respectively (Fig. 4).

A negative CIE of − 5.4 ‰ in δ^13^C_TOC_ is observed in the lowermost limestone units of the core (279–300 m, middle Permian Negative CIE (mPN) 1, Kungurian to Roadinian APP3; Fig. 3), with the lowest values reaching − 28.43 ‰. Two further excursions with values as negative as − 27.33 ‰ (257.6 m; mPN2) and − 27.38 ‰ (225.3 m; mPN3) are identified in the Guadalupian Lower Parmeener glaciomarine deposits (Fig. 3).

## Triassic of N-E tasmania (Induan–Norian)

### Early Triassic (Induan–Olenekian; interval 210.4–217 m)

Early Triassic sediments representing Unit 2 of the Upper Parmeener Supergroup are relatively thin in Bicheno-5 (210–217 m; Fig. 2). This unit unconformably overlies the middle Permian sediments and is disconformably overlain by the upper Ladinian (APT3) Unit 3 Quartz and Lithic Sandstone of the Upper Parmeemer Supergroup. Unit 2 comprises of quartz sand deposited in a fluvial system flowing southeasterly from the highlands of northeast Tasmania^75^. This quartz-rich sandstone sequence and related rock types (Unit 2) were deposited during the Griesbachian to pre-Anisian^74,77^ but contain significant extraclasts and pebbles from the underlying Lower Parmeneer Super Group. The TOC is very low, with values < 0.25% (Fig. 3). Si/Al ratios are comparatively higher than elsewhere in the core with values up to 12.1, while K values are below the detection limit due to the extremely high silica content in this interval (Fig. 4). Aside from silica domination, kaolin and minor carbonates are also present.

### Late middle to Late Triassic (Ladinian–Norian; interval 6.5–210.4 m)

The uppermost c. 210 m of Bicheno-5 represents Unit 3 (Ladinian) and Unit 4 (Carnian–Norian) of the Upper Parmener Supergroup and includes fluvial lithic to volcanolithic sandstones and mudstones with some thin coal units of the Mount Nicholas Coal Measure^77^. A coal bed with a thickness of 1.1 m is also present at the base of unit 4 in the Bicheno-5 core (166.4–167.5 m).

Seven samples for palynological analysis were selected from the intervals of 10.5 m, 36.9 m, 83.4 m, 120.15 m, 154.1 m, 167.4 m, and 200.05 m (Fig. 3). The sample collected from 200.05 m represents an entirely continental assemblage with pollen, spores, phytoclasts, and abundant fragments of leaf cuticles assigned to the corystosperm *Dicroidium*. Based on the presence of the index taxon *Aratrisporites parvispinosus* we refer this sample to the *Aratrisporites parvispinosus* Zone (APT 3). Associated taxa in this sample include *Cyathidites minor*, *Alisporites australis*, *Uvaesporites* spp*.,* and *Acanthotriletes microspinosus*.

The assemblages from samples collected from the interval 10.5–167.4 m clearly show an affinity with the *Craterisporites rotundus* (APT4) Zone containing a typical mid-Late Triassic flora dominated by *Alisporites* spp. chiefly produced by *Dicroidium*. Minor but important elements include the spore taxa *Aratrisporites spinosus, Limbosporites denmeadi* (FAD at 153.3 m), *Cyathidites minor, C. australis, Densoisporites* spp., *Stereisporites* spp., and *Striatella seebergensis*. Pollen taxa occurring in low portions include monosulcate pollen grains possibly produced by Ginkgoales and by ‘seed-ferns’ belonging to the Peltaspermales^5^. This age agrees with the palynostratigraphic zone and age determinations for Unit 3 and 4 from ^77^ on correlative cores FT82B and GY27 for Units 3 and 4 in the Mount Nicholas Coal Measure (Fig. SI–S2). The TOC values in mudstone-dominated deposits (167.4–168.65 m; 154.6–155.7 m; 109–123 m; Fig. 3) are significantly higher than in the sandstone-dominated deposits (eg. 156–166 m; 141.75–153 m; 123.3–128.8 m; Fig. 3), with values up to 8.15% compared to 0.02% in the sandstones, and the C/N ratio ranges from 0.57 to 180.84 (Fig. SI–S4). The Si/Al and K/Al vary, potentially cyclically, with the highest values in the early Norian period (i.e., ~ 80–100 m; Fig. 4).

The δ^13^C_TOC_ in the Carnian age sediments of the core (141.3–169 m; Fig. 3) shows a negative CIE (CN) with four marked steps of − 27.59 ‰ (CN1, 164.55 m), − 27.8 ‰ (CN2, 161.9 m), − 28.51 ‰ (CN3, 157.05 m), and -27.32 ‰ (CN4, 141.8 m). CN3 is marked by an abrupt positive shift of 3.4 ‰, which correlates with an abrupt change in lithology from lithic sandstone to mudstone. The final excursion CN4 represents an interval of significant variability in the isotope values with values ranging from − 27.32 to − 24.06 ‰, which also coincides with a lithological change to more mud-dominated deposits. This interval is accompanied by a marked increase in TOC with values up to 8% (Fig. 3). Si/Al and K/Al show significant shifts at the base of the CPE, specifically in CN1, where a marked increase in Si/Al and Ti/Al ratio occurs (Fig. 4).

Norian age sediments show a decrease in thin coal deposits and a return to more fluvial sand and overbank mud deposits. This interval is marked by a ~ 2 ‰ negative CIE (mNN, 47.6–66 m; Fig. 3) with values as low as − 28.21 ‰ (64.4 m), although there is no notable change in the TOC during the excursion (~ 1%). TOC values increase following the excursion to > 2% (~ 45 m). In addition to the increase in TOC following the MNE there is a significant change in the Ti/Al with a prolonged decrease in values (Fig. 4), while the δ^13^C_TOC_ returns to more typical background values of − 25‰ with some variability but no significant negative perturbations.

## Discussion

The isotopic composition of marine and terrestrial organic matter is influenced by various factors, including the source of the organic matter, local and regional environmental conditions, and the exchange between global carbon reservoirs^84–86^. Analyzing the ratios of C/N and δ^13^C_TOC_ values can help determine the source of the organic matter. The C/N ratios (Fig. SI–S4) indicate that samples from the Triassic period are more terrestrial than those from the middle Permian, which have relatively lower values. Two specific limestone samples from the Permian section are rich in bio-clast/debris and have significantly higher C/N ratios, which could be due to a terrestrial influx^84,86^. Analysis of C/N and δ^13^C_TOC_ in Bicheno-5 (Fig. SI–S5) reveals that the organic matter found at a depth of 0–217 m in the middle to Late Triassic sediments mostly comes from lacustrine algae and C3 land plants. This result is consistent with the presence of visible macerated plant debris in hand samples and in the macerals seen in the palynological slides. However, the middle Permian (217–300 m) shows variability, indicating a mix of marine and terrestrial sources.

Temporal shifts in δ^13^C_TOC_ values are influenced by changes in the ratio of terrestrial to marine organic matter in the succession^84–86^. However, there is no statistically significant linear relationship (r = 0.19) between δ^13^C_TOC_ and C/N in Bicheno-5, indicating that the primary control on temporal variations in δ^13^C_TOC_ does not come from organic matter sources. It appears that the Bicheno-5 δ^13^C_TOC_ is also not significantly impacted by local or regional environmental conditions. This is because the samples can be directly associated with climatic interpretations based on sedimentologic observations from the same succession. Moreover, δ^13^C_TOC_ shifts from the Bicheno-5 middle Permian occur in line with P3-P4 glacial intervals that have been documented in the same region in the SW Sydney Basin^87^. Therefore, the δ^13^C_TOC_ shifts most likely reflect climate-related changes in carbon cycling in both terrestrial and marine environments, which could be related to changes in atmospheric pCO_2_ caused by an increase in organic carbon burial.

The combined δ^13^C_TOC_ and chronology of Bicheno-5 suggests the presence of three significant CIEs that can be correlated to globally recognized events, namely the Guadalupian middle Permian CIE, the Carnian CIE (associated with the CPE) and the middle Norian CIE. To reduce the variation in δ^13^C_TOC_ due to temporal or interbasinal changes in water availability, water stress, and photosynthesis fractionation, correlation mainly carried out with CIE from the same depositional environment. The extreme seasonality of the high paleolatitude setting of Bicheno-5 may result in a relatively 'noisy' record compared to correlative sections. Therefore, δ^13^C_TOC_ from Bicheno-5 was also displayed using a 2-point moving average approach (Fig. 3).The Gaudalupian CIE (middle Permian) in Bicheno-5 can be correlated with sediments recovered from boreholes in Chaohu, China^13^ (Fig. 3). These boreholes represent radiometrically and palynologically age-controlled high-resolution carbon isotopes and XRF. The sub-cyle of CIE episodes from the late Kungurian (mPN1) and Wordinian (mPN2) are similar in magnitude to those from the Chaohu borehole (~ − 3 ‰), while the Capitanian (mPN3) has a slightly different magnitude. In Bicheno-5, the Capitanian (mPN3) δ^13^C_TOC_ values decrease by 1.75 ‰, to a value of -27.38 ‰, compared to a negative shift of only − 0.75 to ~ − 26 ‰ in the boreholes from the Gufeng Formation Chaohu, China^13^. Similar trends in δ^13^C_TOC_ for this interval have been noted in the Sydney Basin, Australia with values as heavy as − 25.19 ‰ recorded in the Kungurian–Roadian^87^. In Bicheno-5, values of ~ − 27 ‰ are observed in the Kungurian–Roadian, significantly lighter and likely reflecting similar depositional and/or environmental changes as in the Sydney Basin. Both mPN2 and mPN3 coincide with deglaciation episodes from Eastern Australia (P3 and P4 phases^87^), which increased the terrestrial sediment influx to the marine system. The increase in Si, Al, and K content in Bicheno -5 indicates such a change (Fig. 4). A decrease in K/Al ratios is typically indicative of stronger chemical weathering. Al tends to be preserved and enriched due to weathering^88^, while K is enriched after mild chemical weathering, but it is depleted after extreme chemical weathering^89^. The lowering K/Al ratio upward into younger strata observed during both mPN2 and mPN3 shows an increased flux of clay and may indicate a higher degree of chemical weathering on land^90–92^.

The four pronounced CIEs observed in the Carnian of Bicheno-5 (CN1-4, CPE Fig. 3) exhibit a similar magnitude of around 3–5 ‰. The record correlates well in both magnitude of the excursion and steps with the record of the Carnian Pluvial Episode CIEs observed in other continental successions such as the Wiscombe Park (WP) borehole, Devon, UK^33^ (shown in Fig. 3), the Jiyuan Basin North China^93^ and the NW Tethys Marine-Marginal Marine composite^24,25^. Si and Al show a relatively low coefficient correlation (r = 0.012), particularly in the Triassic Sect. (0–217 m) as the influx of sand and clay is independent, typical of a fluvial environment (Fig. 4).

In the Upper Parmeener Supergroup, the elemental ratio is very much affected by the rapid depositional changes from various facies in the fluvial-lacustrine environment such as the channels, bars, oxbows, swamps, etc. Particularly in the base of the CPE section, there is a shift of Si/Al and K/Al ratio but the Ti/Al remains relatively steady. Coal seams first appear at the base of the proposed CPE, silica dominates the fluvial-lacustrine system although following the CPE there is an increase in smectite and chlorite as indicated by HyLogging data. The increase of smectite and the occurrence of thin coal seams may be indicative of more warm-humid conditions that led to increased vegetation and chemical weathering leading to clay formation^94,95^.

The Middle Norian CIE (MNE) has a limited global record thus it is correlated with the relatively thinner pelagic sediments from Sakahogi section, Japan^37^ (Fig. 3). During the MNE (Fig. 4), the increase in Ti/Al is not accompanied by an increase of Si/Al and K/Al, and the log shows no significant increase in more coarse-grained sediment. The stable values of Si/Al and K/Al reflect a relatively stable sandstone-dominated fluvial environment.

The change in Ti content in the mid-Norian may be associated with changes in mineralogy and provenance of the sandstones. Fluvialy reworked volcanolithic sandstone begins to dominate the system indicating the sediment source changed from the Beardmore-Ross Upland to a magmatic arcdue to the foreland basin's westward migration^96^. Although the strata during the MNE are still dominated by volcanolithic sandstone, there is evidence from the HyLogging data of more clay minerals (specifically smectite), likely sourced from distal volcanic ash, above the height of 67 m in comparison to the underlying sediment. It could be indicative of increased chemical weathering with more humid conditions^94,95^. Ti/Al reverts to its normal baseline composition with decreasing smectite, k-feldspar and plagioclase, and dominant silica following the MNE.

## Conclusion

The high-resolution δ^13^C_TOC_ record from N-E Tasmania preserves multiple carbon-isotope excursions spanning across the middle Permian and Late Triassic, as well as evidence of significant climate variability near the paleo-South pole. Three major carbon isotope excursion (CIE) intervals characterized by negative shifts of up to 6 ‰ were recognized; the middle Permian, Late Triassic Carnian, and middle Norian. These CIEs can further be correlated with global δ^13^C_TOC_ records from the paleo-Pacific Ocean (Panthalassa), Southwest England, and South China. These carbon cycle perturbations triggered climatic change and environmental responses, reflected in the sedimentology and weathering proxies. The records reflect the complex interplay between the changing continental configuration of Pangea, driven by the emplacement of Large Igneous Provinces, carbon cycle perturbations and climate. Tasmania in the Permian and Triassic occupied a position today occupied by Antarctica and the Antarctic circle. The record from Bicheno-5 highlights the significant impact on weathering and climate in the paleo-South pole due to significant perturbations of *p*CO_2_. While further studies are needed to better understand the magnitude and duration of both the climatic change and resulting weathering and biotic fluxes during extreme events such as the CPE, especially in high latitudes, the record from the mid-Permian and Late Triassic of Tasmania highlights the potential impacts of ongoing Anthropogenic climate change in the Antarctic.

## Methods

Hylogged and pXRF data were collected by Mineral Resources Tasmania (MRT) Core Library, Mornington, Tasmania in 2021. XRF data were collected every 0.5 m using an Olympus Vanta M Series pXRF. The instrument uses a 4-Watt X-ray tube with application-optimized anode material (rhodium Rh and tungsten W): 8–50 kV with a large area Silicon Drift Detector. The instrument uses the built-in Olympus Vanta analysis software version 3.12.34. It was calibrated against a Jurassic dolerite. Results for SiO_2_, TiO_2_, Al_2_O_3_, and K_2_O are reported in weight percent (wt%), translated into Si, Ti, Al, and K elemental masses and their ratios.

The core was scanned using a HyLogger core scanner and high-resolution digital images were acquired. Samples were taken through the 300 m core at a 50 cm resolution. 418 rock samples were decalcified for organic carbon isotope analysis (cf.^97^). 10 g of powdered samples were acidified in 3 M HCL at 60 °C, then neutralized, dried, and powdered before being sent to Elemtex Labs, UK, for analysis. De-carbonated samples were analyzed using the Thermo Flash EA1112 under Eager software for total carbon and nitrogen content (wt%) and then analyzed using a Sercon Hydra 2022 isotope ratio mass spectrometer linked to a Sercon ANCA elemental analyzer that runs in continuous flow mode. Values were corrected using international and in-house standards and reported relative to Vienna Pee Dee Belemnite (VPDB). Instrument reproducibility was constrained through replicate analysis of L-Glutamine (USGS40 and USGS41), producing a mean value − 26.39 and − 37.63‰ respectively. Replicate analysis showed a precision of ±  < 0.2‰ (2 SD).

Ten samples were selected for palynological analyses from the interval 289.25–10.5 m to provide a biostratigraphical framework. Five grams of sedimentary rock/sample were processed according to standard palynological procedures at Global Geolab Limited, Medicine Hat, Canada, including hydrochloric acid (HCl) and hydrofluoric acid (HF) treatment. Kerogene samples were made from each residue (= no oxidation nor sieving) before low grade oxidation and sieving of the organic residues using a 5 μm mesh was carried out on the rest of the residue before it was mounted in epoxy resin on two slides per sample. The palynomorphs in the slides were analysed with a light microscopy, Olympus Cx-43. One hundred palynomorphs (pollen, spores, and algae) per sample were identified, and the slides were further searched for rare taxa or key species useful for palynostratigraphy.

### Supplementary Information

Supplementary Information 1.

Supplementary Information 2.

## Data Availability

All data generated or analysed during this study are included in this published article [and its supplementary information files].
