# Supplementary material for: Carbon cycle perturbations and environmental change of the middle permian and Late Triassic Paleo-Antarctic circle
Source: Sci Rep. 2024 Apr 28;14:9742. doi: 10.1038/s41598-024-60088-5 (PMC11056376; doi:10.1038/s41598-024-60088-5)
Supplement: Supplementary file 1 — Supplementary Information 1. [file 41598_2024_60088_MOESM1_ESM.pdf]

Table SI-1 Geochemical data of Total Organic Carbon (TOC) and $\delta^{13}C_{TOC}$ from Bicheno-5 core, Tasmania.

Sample Number	Depth Start (m)	Depth end (m)	Sample Depth	TOC (%)	$\delta^{13}C_{TOC}$ (‰)
S1	7	7.2	7.1	1.69	-24.78
S2	8.3	8.6	8.45	0.34	-24.68
S3	9.5	9.7	9.6	0.88	-24.34
S4	10	10.2	10.1	0.36	-25.65
S5	10.5	10.7	10.6	3.17	-25.51
S6	11	11.3	11.15	0.16	-26.04
S7	12	12.3	12.15	0.21	-25.95
S8	13	13.3	13.15	0.14	-25.93
S9	14.3	14.6	14.45	0.07	-26.83
S10	15	15.3	15.15	0.08	-24.83
S11	15.7	15.9	15.8	0.65	-24.86
S12	16.4	16.8	16.6	0.93	-24.65
S13	17	17.2	17.1	0.04	-26.33
S14	17.7	17.9	17.8	0.00	-26.71
S15	18	18.4	18.2	0.18	-26.42
S16	18.7	18.9	18.8	0.20	-25.64
S17	19	19.3	19.15	0.12	-25.77
S18	20	20.2	20.1	0.13	-25.65
S19	20.7	20.9	20.8	0.08	-26.04
S20	21.4	21.8	21.6	2.90	-24.75
S21	22.1	22.3	22.2	0.06	-26.45
S22	22.7	22.9	22.8	0.11	-25.47
S23	23.3	23.5	23.4	0.13	-25.30
S24	24.2	24.5	24.35	0.21	-24.86
S25	24.8	25	24.9	0.07	-26.48
S26	25.2	25.5	25.35	0.46	-25.34
S27	26	26.3	26.15	0.22	-25.58
S28	26.6	26.8	26.7	0.11	-26.19
S29	27	27.5	27.25	0.05	-26.46
S31	29	29.3	29.15	0.94	-25.59
S32	29.5	29.8	29.65	3.86	-25.67
S33	30.3	30.5	30.4	1.01	-26.17
S34	30.8	31	30.9	0.73	-25.39
S39	34.2	34.5	34.35	1.00	-25.82

S40	34.6	34.8	34.7	0.10	-26.49
S41	35.2	35.5	35.35	0.11	-25.69
S42	35.8	36	35.9	0.11	-25.48
S43	36.3	36.6	36.45	0.10	-26.22
S44	36.9	37.1	37	1.05	-25.48
S45	37.6	37.7	37.65	0.66	-25.61
S46	38.3	38.7	38.5	0.59	-25.97
S47	38.9	39.2	39.05	0.41	-26.05
S48a	39.6	39.8	39.7	0.03	-26.62
S48b	40	40.4	40.2	0.23	-26.12
S49	41	41.2	41.1	0.76	-25.15
S50	41.6	41.9	41.75	0.10	-25.94
S51	42	42.2	42.1	0.16	-25.85
S52	42.4	42.6	42.5	0.59	-25.30
S53	43	43.5	43.25	0.52	-25.61
S54	43.8	43.9	43.85	0.22	-25.98
S55	44	44.1	44.05	0.36	-26.70
S56	44.7	44.9	44.8	0.23	-25.88
S57	45.1	45.3	45.2	0.39	-25.54
S58a	45.6	45.9	45.75	0.67	-25.37
S58b	46	46.1	46.05	0.07	-26.52
S59	46.5	46.7	46.6	0.62	-25.56
S60	47	47.1	47.05	0.20	-26.13
S61	47.5	47.7	47.6	0.07	-26.12
S62	48.1	48.2	48.15	0.04	-27.44
S63	48.5	48.7	48.6	0.12	-25.43
S64	49.1	49.3	49.2	0.10	-26.65
S65	49.7	49.9	49.8	0.59	-25.13
S66	50.2	50.4	50.3	0.27	-26.49
S67	50.7	50.8	50.75	0.09	-26.91
S68	51.1	51.3	51.2	0.11	-26.76
S69	51.6	51.8	51.7	0.05	-27.02
S70	52.2	52.5	52.35	0.07	-27.51
S71	52.8	53	52.9	0.08	-26.54
S72	53.3	53.5	53.4	0.05	-26.09
S73	53.8	54.1	53.95	0.07	-27.36
S74	54.3	54.5	54.4	0.03	-27.01
S75	54.8	55.1	54.95	0.05	-26.37
S76	55.2	55.4	55.3	0.10	-26.06
S77	55.8	56	55.9	0.08	-26.29

S78	56.3	56.5	56.4	0.04	-27.61
S79	56.8	57	56.9	0.05	-25.60
S80	57.2	57.4	57.3	0.09	-25.83
S81	57.8	58	57.9	1.31	-24.69
S82	58.2	58.4	58.3	0.02	-27.39
S83	59	59.2	59.1	0.69	-25.18
S84	59.5	59.7	59.6	0.12	-26.05
S85	59.9	60.2	60.05	0.05	-26.54
S86	60.5	60.7	60.6	0.03	-27.56
S87	61	61.2	61.1	0.07	-25.89
S88	61.3	61.5	61.4	0.12	-26.54
S89	61.8	62	61.9	0.10	-26.39
S91	62.8	63	62.9	0.18	-25.72
S92	63.3	63.5	63.4	0.10	-26.15
S93	63.8	64	63.9	0.11	-26.68
S94	64.3	64.5	64.4	0.04	-28.21
S95	64.8	65	64.9	0.07	-26.37
S96	65.4	65.6	65.5	0.06	-26.62
S97	65.9	66.1	66	0.07	-25.77
S98	66.4	66.6	66.5	0.02	-27.35
S99	66.9	67.1	67	0.07	-27.04
S100	67.3	67.5	67.4	0.09	-26.07
S101	67.8	68	67.9	0.08	-26.78
S102	68.4	68.6	68.5	0.01	-27.03
S103	69.8	69.2	69.5	0.08	-26.47
S104	69.3	69.5	69.4	0.15	-26.66
S105	69.9	70.2	70.05	0.53	-25.09
S106	70.4	70.6	70.5	0.02	-26.64
S107	71	71.2	71.1	0.08	-25.44
S108	71.6	71.8	71.7	0.15	-25.59
S109	72	72.2	72.1	0.06	-26.53
S110a	72.4	72.5	72.45	0.06	-27.08
S110b	72.75	72.85	72.8	0.07	-26.51
S110c	73.1	73.2	73.15	0.07	-26.60
S111	73.5	73.7	73.6	0.07	-26.95
S112	73.9	74.1	74	0.04	-25.63
S113	74.35	74.55	74.45	0.06	-26.59
S114	74.9	75.1	75	0.16	-25.45
S115a	75.4	75.6	75.5	0.07	-26.72
S115b	75.75	75.95	75.85	0.18	-25.81

S115c	76.1	76.3	76.2	0.09	-26.04
S116	76.5	76.7	76.6	0.14	-26.90
S117	76.9	77.1	77	0.16	-25.94
S118	77.4	77.6	77.5	0.02	-26.06
S119	77.9	78.1	78	0.08	-27.29
S120	78.4	78.6	78.5	0.09	-25.81
S121	79	79.2	79.1	0.06	-26.52
S122	79.5	79.7	79.6	0.04	-25.72
S123	79.9	80.1	80	0.07	-26.41
S124	80.5	80.7	80.6	0.06	-26.48
S125	81	81.2	81.1	0.06	-26.31
S126a	81.45	81.55	81.5	0.03	-26.69
S126b	81.9	82	81.95	0.08	-25.89
S127	82.3	82.5	82.4	0.12	-24.69
S128	82.9	83.1	83	0.05	-26.47
S129	83.4	83.6	83.5	0.13	-23.62
S130	83.9	84.2	84.05	0.13	-25.93
S131a	84.5	84.6	84.55	0.11	-26.07
S131b	85	85.1	85.05	0.10	-26.46
S132a	85.5	85.6	85.55	0.04	-26.50
S132b	86	86.2	86.1	0.10	-25.79
S133	86.3	86.5	86.4	0.13	-26.59
S135a	87.5	87.7	87.6	0.31	-25.44
S135b	88.15	88.25	88.2	0.36	-24.66
S136	88.4	88.6	88.5	0.23	-25.14
S137	89.1	89.3	89.2	0.21	-25.36
S138	89.5	89.6	89.55	0.01	-24.85
S139	89.9	90.1	90	0.00	-26.29
S140	90.5	90.7	90.6	0.61	-24.79
S141	91.05	91.25	91.15	0.13	-23.70
S142	91.6	91.8	91.7	0.15	-25.26
S143	92	92.2	92.1	0.14	-24.37
S144a	92.5	92.6	92.55	0.07	-25.53
S144b	92.7	92.8	92.75	0.08	-26.71
S145	93.4	93.6	93.5	0.07	-25.43
S146	93.8	94	93.9	0.03	-25.44
S147	94.35	94.55	94.45	0.85	-24.51
S148	94.8	95	94.9	0.18	-25.39
S149	95.3	95.5	95.4	0.15	-25.22
S150	95.8	96	95.9	0.05	-24.79

S151a	96.5	96.6	96.55	0.08	-26.04
S151b	97.1	97.2	97.15	0.08	-26.81
S152	97.3	97.5	97.4	0.07	-26.74
S153a	97.65	97.85	97.75	0.02	-25.91
S153b	98.1	98.2	98.15	0.07	-25.47
S154a	98.3	98.4	98.35	0.95	-23.69
S155a	98.6	98.7	98.65	0.67	-25.18
S155b	99.1	99.2	99.15	0.04	-24.92
S156	99.3	99.5	99.4	0.10	-25.72
S157	99.9	100.1	100	0.25	-24.49
S158	100.4	100.6	100.5	0.06	-26.18
S159	101	101.2	101.1	0.36	-24.21
S160	101.6	101.8	101.7	0.05	-26.07
S161	102	102.2	102.1	0.21	-24.87
S162	102.5	102.7	102.6	0.36	-25.17
S163	103	103.2	103.1	0.10	-24.94
S164	103.5	103.7	103.6	0.16	-25.59
S165	104	104.2	104.1	0.08	-25.55
S166	104.4	104.5	104.45	0.07	-25.97
S167	104.9	105	104.95	0.02	-25.86
S168	105.4	105.5	105.45	0.51	-25.22
S169	105.9	106.1	106	0.14	-24.95
S171	106.9	107.1	107	0.19	-25.34
S172	107.5	107.7	107.6	0.25	-25.77
S173	108.1	108.3	108.2	0.51	-25.31
S174	108.7	108.9	108.8	0.12	-26.22
S175	109.1	109.2	109.15	0.35	-25.51
S176	109.5	109.7	109.6	0.26	-25.65
S177	109.95	110.05	110	4.19	-25.20
S178	110.55	110.65	110.6	0.82	-25.54
S179	111	111.2	111.1	0.76	-24.72
S180	111.5	111.7	111.6	0.87	-25.53
S181	112.2	112.4	112.3	0.75	-25.27
S182	112.8	113	112.9	0.65	-25.36
S183	113.6	113.6	113.6	0.69	-25.72
S184	114.1	114.1	114.1	0.56	-25.49
S185	114.7	114.8	114.75	3.51	-24.46
S186	115.2	115.3	115.25	0.30	-25.64
S188	116.2	116.3	116.25	0.68	-25.33
S189	116.8	116.9	116.85	6.67	-25.70

S192	118.2	118.4	118.3	0.49	-24.34
S193	118.75	118.85	118.8	0.37	-26.18
S194	119.15	119.25	119.2	0.35	-24.28
S195	119.7	119.8	119.75	2.07	-26.07
S196	120.15	120.25	120.2	3.27	-25.26
S197	120.6	120.7	120.65	3.42	-25.70
S198	121.3	121.4	121.35	0.54	-26.14
S199	121.75	121.85	121.8	0.63	-26.22
S200	122.2	122.3	122.25	0.41	-25.57
S201	122.6	122.7	122.65	0.28	-25.93
S202	122.9	123	122.95	0.87	-24.74
S203	123.3	123.4	123.35	0.09	-26.41
S204	123.6	123.7	123.65	0.08	-25.48
S205	124.1	124.2	124.15	0.09	-26.85
S206	124.7	124.9	124.8	0.07	-26.13
S207	125.3	125.4	125.35	0.08	-26.55
S208	125.7	125.8	125.75	0.02	-26.60
S209	126.2	126.3	126.25	0.04	-26.08
S210	126.7	126.8	126.75	0.28	-24.13
S211	127.2	127.4	127.3	0.12	-26.13
S212	127.7	127.8	127.75	0.06	-25.87
S213	128.1	128.2	128.15	0.05	-26.08
S214	128.7	128.8	128.75	0.07	-26.89
S215	129.1	129.4	129.25	3.21	-25.40
S216	129.4	129.5	129.45	1.73	-25.35
S217	129.7	129.9	129.8	0.08	-26.64
S218	130.2	130.4	130.3	0.12	-24.88
S219	130.6	130.7	130.65	0.05	-25.76
S220	131.1	131.2	131.15	0.08	-25.11
S221	131.7	131.8	131.75	0.61	-25.87
S222	132.1	132.3	132.2	0.06	-26.21
S223	132.9	133.1	133	0.05	-26.53
S225	133.8	134	133.9	0.05	-26.21
S226	134.4	134.5	134.45	0.12	-25.49
S227	134.8	134.9	134.85	0.80	-25.11
S229	135.8	135.9	135.85	0.89	-25.26
S230	136.3	136.4	136.35	0.69	-25.42
S231	136.7	136.8	136.75	0.91	-25.63
S232	137.15	137.25	137.2	0.61	-25.20
S233	137.65	137.75	137.7	0.46	-25.61

S234	138	138.1	138.05	0.43	-24.62
S235	138.4	138.5	138.45	0.49	-25.67
S236	138.7	138.8	138.75	0.73	-25.50
S237	139.2	139.3	139.25	0.56	-25.69
S238	139.4	139.5	139.45	0.68	-25.53
S239	139.75	139.85	139.8	0.41	-25.74
S240	140.3	140.4	140.35	0.26	-25.88
S241	140.65	140.85	140.75	0.67	-25.65
S242	141.2	141.4	141.3	0.35	-24.06
S243	141.75	141.85	141.8	0.06	-27.32
S244	142.3	142.4	142.35	0.04	-25.42
S245	142.8	143	142.9	0.07	-27.20
S246	143.35	143.55	143.45	0.19	-25.27
S247	143.9	144	143.95	0.08	-27.10
S248	144.4	144.5	144.45	0.04	-26.47
S249	144.8	144.9	144.85	0.06	-26.66
S250	145.3	145.3	145.3	0.18	-25.46
S251	145.9	146	145.95	0.05	-27.15
S252	146.4	146.6	146.5	0.28	-24.82
S253	146.85	146.95	146.9	0.09	-27.13
S254	147.35	147.45	147.4	0.06	-26.54
S255	148.2	148.4	148.3	0.17	-26.39
S256	148.85	148.95	148.9	1.01	-25.88
S257	149.05	149.15	149.1	0.07	-27.11
S258	149.4	149.6	149.5	0.08	-26.31
S259	149.8	150	149.9	0.59	-25.60
S260	150.1	150.3	150.2	0.20	-24.84
S261	150.6	150.7	150.65	0.06	-26.44
S262	151.2	151.4	151.3	0.09	-26.74
S263	151.7	151.8	151.75	0.22	-25.90
S264	152.1	152.2	152.15	0.16	-26.27
S265	152.6	152.7	152.65	0.12	-26.51
S266	153.05	153.15	153.1	0.14	-26.59
S267	153.65	153.75	153.7	0.06	-26.76
S268	154.1	154.3	154.2	7.64	-25.01
S269	154.6	154.8	154.7	4.54	-25.17
S270	155.3	155.4	155.35	0.21	-25.40
S271	155.7	155.7	155.7	6.27	-25.09
S272	156.1	156.2	156.15	0.04	-27.29
S273	156.4	156.5	156.45	0.09	-27.05

S274	157	157.1	157.05	0.17	-28.51
S275	157.5	157.6	157.55	0.08	-27.09
S276	158.1	158.2	158.15	0.06	-27.06
S277	158.42	158.52	158.47	0.07	-26.83
S278	158.95	159.05	159	0.07	-26.50
S279	159.4	159.5	159.45	0.09	-26.52
S280	160	160.1	160.05	0.04	-27.04
S281	160.7	160.8	160.75	0.09	-26.43
S282	161	161.1	161.05	0.15	-27.03
S283	161.4	161.5	161.45	0.11	-26.73
S284	161.8	162	161.9	0.03	-27.80
S285	162.4	162.5	162.45	0.14	-26.15
S286	162.95	163.05	163	0.07	-26.34
S287	163.4	163.5	163.45	0.21	-25.33
S288	163.9	164	163.95	0.03	-27.22
S289	164.1	164.2	164.15	0.06	-26.68
S290	164.5	164.6	164.55	0.15	-27.59
S291	164.9	165	164.95	0.07	-26.85
S292	165.45	165.55	165.5	0.04	-26.87
S293	166	166.2	166.1	0.07	-26.63
S294	166.35	166.55	166.45	0.60	-25.22
S296	167.4	167.5	167.45	3.62	-26.12
S297	167.9	168	167.95	4.78	-25.10
S298	168.2	168.3	168.25	8.15	-25.99
S299	168.55	168.65	168.6	2.04	-25.42
S300	169.1	169.2	169.15	1.26	-25.11
S302	170	170.1	170.05	0.06	-25.78
S306	172.1	172.2	172.15	5.92	-25.65
S308	173	173.2	173.1	3.92	-25.68
S310	174	174.1	174.05	0.14	-26.06
S312	174.9	175.1	175	0.12	-26.93
S314	175.85	175.95	175.9	0.18	-24.24
S316	177.1	177.3	177.2	0.05	-26.99
S318	178.2	178.3	178.25	0.08	-26.75
S320	179.3	179.5	179.4	0.19	-25.76
S322	180.5	180.7	180.6	0.12	-25.40
S324	181.7	181.8	181.75	0.36	-24.61
S327	182.9	183	182.95	1.03	-25.32
S329	183.9	184.1	184	0.91	-24.87
S331	185	185.1	185.05	0.38	-25.32

S333	185.7	185.8	185.75	0.15	-26.44
S335	186.4	186.6	186.5	0.16	-26.01
S337	187.5	187.7	187.6	7.77	-25.75
S341	189.9	190.1	190	8.70	-24.15
S352	196	196.1	196.05	0.24	-26.34
S354	197.1	197.2	197.15	0.18	-25.87
S356	198.2	198.3	198.25	0.13	-26.36
S358	199.1	199.2	199.15	0.61	-26.14
S360	200.05	200.15	200.1	4.06	-25.84
S362	201.05	201.15	201.1	7.29	-25.51
S364	202.05	202.15	202.1	0.42	-26.00
S366	202.9	203	202.95	0.55	-26.82
S368	203.9	204	203.95	3.50	-26.07
S370	204.9	205	204.95	0.49	-25.56
S372	206.1	206.3	206.2	0.46	-26.58
S374	207.1	207.2	207.15	0.59	-26.15
S382	209.9	210.1	210	5.36	-26.46
S384	211	211.1	211.05	0.04	-26.44
S386	211.9	212.1	212	0.12	-25.76
S388	212.7	212.9	212.8	0.04	-26.29
S390	213.5	213.7	213.6	0.20	-26.26
S392	214.7	214.8	214.75	0.00	-25.60
S395	216.3	216.5	216.4	0.04	-26.77
S397	217.5	217.6	217.55	0.12	-25.24
S399	218.4	218.5	218.45	0.69	-27.44
S401	219.4	219.5	219.45	0.63	-26.25
S403	220.3	220.4	220.35	0.61	-25.48
S405	221.3	221.4	221.35	0.81	-25.75
S407	222.4	222.5	222.45	0.57	-25.79
S409	223.5	223.6	223.55	0.13	-26.86
S411	224.35	224.45	224.4	0.66	-27.09
S413	225.15	225.35	225.25	0.40	-27.38
S415	226.3	226.4	226.35	0.51	-27.07
S417	227.3	227.4	227.35	0.40	-26.05
S419	228.3	228.4	228.35	0.49	-27.21
S421	229.3	229.4	229.35	0.44	-27.21
S423	230.3	230.4	230.35	0.40	-25.08
S425	231.3	231.4	231.35	0.58	-26.69
S427	232.3	232.4	232.35	0.51	-25.49
S431	234.3	234.4	234.35	0.51	-25.77

S433	235.3	235.4	235.35	0.41	-25.56
S435	236.3	236.4	236.35	0.43	-25.38
S437	237.3	237.4	237.35	0.39	-25.08
S439	238.1	238.2	238.15	0.29	-24.42
S441	238.8	238.9	238.85	0.61	-24.99
S443	239.8	239.9	239.85	0.40	-25.51
S445	240.6	240.8	240.7	0.42	-25.24
S447	241.5	241.7	241.6	0.50	-25.66
S449	242.6	242.7	242.65	0.36	-24.58
S451	243.3	243.4	243.35	0.53	-25.36
S453	244.3	244.4	244.35	0.41	-26.31
S455	245.4	245.5	245.45	0.77	-27.03
S457	246.8	247	246.9	0.71	-24.85
S459	247.8	247.9	247.85	0.77	-25.73
S461	249.1	249.3	249.2	1.02	-26.30
S463	250.95	251.05	251	0.75	-27.01
S465	251.9	252	251.95	1.31	-25.22
S467	252.9	253	252.95	0.80	-26.09
S469	253.8	253.9	253.85	0.88	-26.36
S471	254.8	255	254.9	0.47	-26.95
S473	256	256.1	256.05	1.22	-25.95
S477	257.8	257.9	257.85	1.24	-27.33
S479	258.8	259	258.9	0.49	-27.30
S481	259.9	260	259.95	0.40	-24.57
S483	260.75	260.85	260.8	0.34	-25.51
S485	261.8	262	261.9	0.14	-25.82
S487	262.95	263.05	263	0.45	-26.74
S489	263.35	263.55	263.45	0.42	-24.60
S491	264.2	264.3	264.25	0.28	-25.16
S493	264.7	264.9	264.8	0.52	-23.02
S495	265.7	265.8	265.75	0.10	-27.15
S497	267	267.1	267.05	0.12	-26.52
S500	268.3	268.5	268.4	0.58	-25.12
S502	269.4	269.5	269.45	0.16	-24.35
S504	270.2	270.3	270.25	0.36	-26.48
S506	271.1	271.2	271.15	0.26	-23.01
S508	272.1	272.4	272.25	0.32	-24.57
S510	273.3	273.4	273.35	0.55	-24.65
S512	274.1	274.3	274.2	0.41	-27.02
S514	275.25	275.35	275.3	0.36	-25.07

S516	276.2	276.3	276.25	0.57	-25.52
S518	277.3	277.5	277.4	0.54	-24.80
S520	278.2	278.3	278.25	0.38	-25.06
S522	279.2	279.3	279.25	0.26	-23.19
S524	280.2	280.3	280.25	0.30	-24.17
S526	281.2	281.3	281.25	0.34	-25.16
S528	282.1	282.3	282.2	0.42	-24.58
S530	283.2	283.4	283.3	0.23	-25.93
S532	284.2	284.3	284.25	0.19	-25.54
S534	285.2	285.3	285.25	0.02	-26.93
S538	287.1	287.2	287.15	0.24	-27.76
S540	288.1	288.2	288.15	0.65	-26.75
S542	289.1	289.25	289.175	0.81	-25.45
S544	290.2	290.3	290.25	0.18	-28.43
S546	291.2	291.3	291.25	0.07	-24.95
S548	292.2	292.3	292.25	0.08	-26.26
S550	293.2	293.3	293.25	0.04	-26.82
S552	294.2	294.3	294.25	0.25	-27.39
S554	295.25	295.53	295.39	0.10	-26.45
S556	296.25	296.35	296.3	0.15	-26.27
S558	297.2	297.3	297.25	0.36	-25.24
S560	298.2	298.3	298.25	0.46	-26.94
S562	299.25	299.35	299.3	0.25	-24.49
S564	300.35	300.55	300.45	0.66	-25.81

Table SI-2 Geochemical pXRF data of SiO_2, K_2O, Al_2O_3, and TiO_2 with the instrument's minimum detection limit. Analysis is not conducted on values below the detection limit (in brackets). Geochemical pXRF data are translated into Si, Ti, Al, and K elemental masses and their Si/Al, K/Al, and Ti/Al ratios.

Sample Depth (m)	SiO2 (%)	K2O (%)	Al2O3 (%)	TiO2 (%)	Si	K	Al	Ti	Si/Al	K/Al	Ti/Al
Detection Limit →	*9.00*	*2.00*	*5.00*	*0.20*							
6.5	68.01	5.98	14.47	1.74	31.76	4.96	7.65	1.04	4.15	0.65	0.14
7	63.74	6.46	14.74	2.48	29.77	5.36	7.80	1.48	3.82	0.69	0.19
7.5	61.29	6.24	13.28	2.23	28.62	5.18	7.03	1.34	4.07	0.74	0.19
8	59.58	5.15	11.92	1.60	27.83	4.27	6.31	0.96	4.41	0.68	0.15
8.5	63.46	6.01	14.94	2.75	29.63	4.99	7.91	1.65	3.75	0.63	0.21
9	56.34	4.68	11.37	1.68	26.31	3.88	6.02	1.01	4.37	0.64	0.17
9.5	60.24	3.92	9.12	1.40	28.13	3.25	4.82	0.84	5.83	0.67	0.17
10	60.08	4.02	9.92	1.42	28.06	3.34	5.25	0.85	5.35	0.64	0.16
10.5	65.00	4.68	10.83	1.30	30.35	3.88	5.73	0.78	5.30	0.68	0.14
11	56.72	4.47	10.54	2.66	26.49	3.71	5.58	1.59	4.75	0.67	0.29
11.5	58.48	4.53	10.81	2.45	27.31	3.76	5.72	1.47	4.77	0.66	0.26
12	61.89	4.62	11.83	2.54	28.90	3.84	6.26	1.52	4.62	0.61	0.24
12.5	68.53	5.34	13.06	2.40	32.00	4.43	6.91	1.44	4.63	0.64	0.21
13	59.26	4.47	11.82	3.02	27.68	3.71	6.25	1.81	4.43	0.59	0.29
13.5	65.63	5.11	12.32	2.41	30.65	4.24	6.52	1.45	4.70	0.65	0.22
14	60.13	4.40	9.84	2.39	28.08	3.65	5.20	1.43	5.40	0.70	0.28
14.5	65.52	4.62	12.11	5.66	30.60	3.84	6.40	3.39	4.78	0.60	0.53
15	57.42	4.19	10.26	2.29	26.82	3.48	5.43	1.37	4.94	0.64	0.25
15.5	67.35	5.11	13.06	2.36	31.45	4.24	6.91	1.41	4.55	0.61	0.20
16	45.29	4.20	9.77	3.41	21.15	3.48	5.17	2.05	4.09	0.67	0.40
16.5	63.71	5.89	13.02	3.39	29.75	4.89	6.89	2.03	4.32	0.71	0.29
17	57.58	3.66	9.89	1.88	26.89	3.04	5.23	1.13	5.14	0.58	0.22
17.5	60.67	4.32	11.05	2.09	28.33	3.59	5.84	1.25	4.85	0.61	0.21
18	56.54	3.73	9.05	2.02	26.40	3.10	4.79	1.21	5.51	0.65	0.25
18.5	67.22	5.79	12.33	2.08	31.39	4.80	6.52	1.25	4.81	0.74	0.19
19	57.80	4.55	10.07	2.30	26.99	3.78	5.32	1.38	5.07	0.71	0.26
19.5	53.72	3.67	7.94	1.94	25.09	3.04	4.20	1.16	5.98	0.72	0.28
20	54.44	3.87	13.07	2.24	25.43	3.22	6.92	1.34	3.68	0.46	0.19
20.5	64.14	4.59	11.24	2.34	29.95	3.81	5.94	1.41	5.04	0.64	0.24
21	63.34	4.59	12.81	2.89	29.58	3.81	6.77	1.73	4.37	0.56	0.26

21.5	64.57	4.42	10.71	2.30	30.15	3.67	5.67	1.38	5.32	0.65	0.24
22	66.29	4.67	12.94	2.14	30.96	3.87	6.85	1.29	4.52	0.57	0.19
22.5	69.00	5.12	11.98	2.44	32.22	4.25	6.34	1.46	5.08	0.67	0.23
23	66.89	5.43	12.13	2.59	31.24	4.51	6.42	1.55	4.87	0.70	0.24
23.5	53.94	3.95	13.00	2.89	25.19	3.28	6.88	1.73	3.66	0.48	0.25
24	64.23	4.91	12.97	1.89	29.99	4.08	6.86	1.13	4.37	0.59	0.17
24.5	66.48	4.40	11.70	2.00	31.05	3.66	6.19	1.20	5.02	0.59	0.19
25	60.15	3.74	9.20	2.61	28.09	3.10	4.87	1.56	5.77	0.64	0.32
25.5	64.21	4.43	13.05	2.47	29.99	3.68	6.90	1.48	4.34	0.53	0.21
26	65.59	4.24	10.59	2.02	30.63	3.52	5.60	1.21	5.47	0.63	0.22
26.5	61.05	3.89	10.50	2.31	28.51	3.23	5.55	1.38	5.13	0.58	0.25
27	45.45	3.10	18.89	2.35	21.23	2.57	10.00	1.41	2.12	0.26	0.14
27.5	34.74	*(0.9)*	15.57	0.86	16.22		8.24	0.51	1.97		0.06
28	15.45	*(0.3)*	6.75	0.37	7.21		3.57	0.22	2.02		0.06
28.5	33.83	*(0.3)*	18.34	1.35	15.80		9.70	0.81	1.63		0.08
29	64.99	5.49	13.12	2.42	30.35	4.56	6.94	1.45	4.37	0.66	0.21
29.5	61.96	6.37	13.36	2.58	28.94	5.28	7.07	1.54	4.10	0.75	0.22
30	68.57	6.06	13.61	2.60	32.02	5.03	7.20	1.56	4.45	0.70	0.22
31	63.34	5.83	13.33	2.43	29.58	4.84	7.05	1.46	4.20	0.69	0.21
33.5	61.40	5.73	11.60	2.47	28.68	4.75	6.13	1.48	4.68	0.78	0.24
34	59.14	6.27	13.41	2.58	27.62	5.20	7.09	1.55	3.89	0.73	0.22
34.5	56.88	4.10	10.15	1.95	26.57	3.40	5.37	1.17	4.95	0.63	0.22
35	68.43	4.05	10.84	1.88	31.96	3.36	5.73	1.13	5.57	0.59	0.20
35.5	62.43	3.83	9.41	2.03	29.16	3.18	4.98	1.21	5.86	0.64	0.24
36	66.92	3.50	9.30	3.67	31.25	2.90	4.92	2.20	6.35	0.59	0.45
36.5	61.07	4.00	9.78	1.63	28.52	3.32	5.17	0.98	5.51	0.64	0.19
37	64.72	4.67	10.29	1.68	30.23	3.87	5.44	1.01	5.55	0.71	0.18
37.5	61.94	4.54	9.61	1.75	28.93	3.77	5.08	1.05	5.69	0.74	0.21
38	67.16	3.97	9.93	2.34	31.36	3.30	5.25	1.40	5.97	0.63	0.27
38.5	66.90	5.09	11.38	2.28	31.24	4.22	6.02	1.36	5.19	0.70	0.23
39	63.43	4.61	9.82	1.82	29.62	3.83	5.19	1.09	5.70	0.74	0.21
39.5	72.85	4.85	10.68	4.58	34.02	4.02	5.65	2.74	6.02	0.71	0.49
40	70.80	5.09	11.36	2.24	33.06	4.22	6.01	1.34	5.50	0.70	0.22
40.5	69.63	4.49	10.12	2.12	32.52	3.72	5.36	1.27	6.07	0.70	0.24
41	69.35	4.67	9.93	1.94	32.39	3.87	5.25	1.16	6.16	0.74	0.22
41.5	68.94	4.92	10.83	2.02	32.19	4.08	5.73	1.21	5.62	0.71	0.21
42	67.04	4.59	10.20	2.07	31.31	3.81	5.39	1.24	5.80	0.71	0.23
42.5	72.85	4.97	10.81	2.29	34.02	4.13	5.72	1.37	5.95	0.72	0.24
43	64.10	5.86	11.60	2.63	29.94	4.86	6.14	1.58	4.88	0.79	0.26
43.5	67.61	5.71	12.47	2.59	31.57	4.74	6.59	1.55	4.79	0.72	0.24

44	70.35	4.91	11.61	2.39	32.85	4.08	6.14	1.43	5.35	0.66	0.23
44.5	66.44	4.88	9.95	1.84	31.03	4.05	5.26	1.11	5.90	0.77	0.21
45	67.97	5.00	9.65	2.03	31.74	4.15	5.11	1.21	6.22	0.81	0.24
45.5	69.36	5.16	11.60	2.76	32.39	4.29	6.13	1.65	5.28	0.70	0.27
46	62.19	4.30	8.36	1.78	29.04	3.57	4.42	1.07	6.57	0.81	0.24
46.5	57.19	4.06	8.68	2.12	26.71	3.37	4.59	1.27	5.81	0.73	0.28
47	48.07	4.24	9.18	2.89	22.45	3.52	4.86	1.73	4.62	0.72	0.36
47.5	50.07	5.85	13.45	3.67	23.38	4.85	7.11	2.20	3.29	0.68	0.31
48	51.40	4.05	9.84	6.74	24.01	3.36	5.21	4.04	4.61	0.65	0.78
48.5	58.16	5.30	12.29	2.69	27.16	4.40	6.50	1.61	4.18	0.68	0.25
49	61.81	5.72	11.76	2.66	28.86	4.75	6.22	1.59	4.64	0.76	0.26
49.5	56.27	6.60	14.01	3.11	26.28	5.48	7.41	1.86	3.55	0.74	0.25
50	58.44	5.57	10.61	3.79	27.29	4.62	5.61	2.27	4.86	0.82	0.40
50.5	59.02	5.23	11.15	3.89	27.56	4.34	5.90	2.33	4.67	0.74	0.40
51	56.06	4.97	12.61	4.52	26.18	4.13	6.67	2.71	3.92	0.62	0.41
51.5	57.37	4.93	11.08	3.01	26.79	4.09	5.86	1.80	4.57	0.70	0.31
52	59.67	4.73	11.19	2.73	27.87	3.93	5.92	1.63	4.71	0.66	0.28
52.5	56.48	4.94	10.78	3.36	26.37	4.10	5.71	2.01	4.62	0.72	0.35
53	62.80	5.09	11.27	2.57	29.33	4.22	5.96	1.54	4.92	0.71	0.26
53.5	55.97	4.58	9.86	2.69	26.14	3.80	5.22	1.61	5.01	0.73	0.31
54	55.92	5.28	10.11	2.98	26.12	4.38	5.35	1.78	4.88	0.82	0.33
54.5	59.63	5.04	10.55	2.73	27.85	4.19	5.58	1.63	4.99	0.75	0.29
55	56.31	4.75	9.80	2.25	26.30	3.94	5.19	1.35	5.07	0.76	0.26
55.5	62.31	6.33	11.09	2.52	29.10	5.25	5.86	1.51	4.96	0.90	0.26
56	61.41	5.68	11.10	2.61	28.68	4.72	5.87	1.57	4.89	0.80	0.27
56.5	59.55	4.60	11.02	3.40	27.81	3.82	5.83	2.04	4.77	0.66	0.35
57	60.18	4.67	10.33	3.18	28.10	3.88	5.47	1.91	5.14	0.71	0.35
57.5	55.60	5.25	9.76	3.18	25.96	4.36	5.16	1.91	5.03	0.84	0.37
58	63.88	5.18	10.76	2.73	29.83	4.30	5.69	1.64	5.24	0.75	0.29
58.5	61.20	6.11	11.28	2.62	28.58	5.07	5.97	1.57	4.79	0.85	0.26
59	57.01	5.59	10.94	2.40	26.62	4.64	5.79	1.44	4.60	0.80	0.25
59.5	57.78	5.45	10.90	2.65	26.98	4.53	5.77	1.59	4.68	0.78	0.28
60	59.84	5.67	11.21	2.74	27.94	4.71	5.93	1.64	4.71	0.79	0.28
60.5	60.03	5.58	10.32	2.47	28.04	4.63	5.46	1.48	5.13	0.85	0.27
61	59.70	5.61	10.77	3.00	27.88	4.66	5.70	1.80	4.89	0.82	0.31
61.5	60.93	5.57	10.43	3.17	28.46	4.63	5.52	1.90	5.16	0.84	0.34
62	60.02	5.38	10.91	2.44	28.03	4.47	5.77	1.46	4.86	0.77	0.25
62.5	59.44	5.31	10.27	2.84	27.76	4.41	5.43	1.70	5.11	0.81	0.31
63	62.07	5.97	11.16	2.56	28.99	4.96	5.91	1.53	4.91	0.84	0.26
63.5	60.11	5.59	10.27	2.96	28.07	4.64	5.44	1.77	5.17	0.85	0.33

64	25.62	*(1.9)*	5.14	2.51	11.97		2.72	1.50	4.40		0.55
64.5	59.36	5.21	10.50	2.70	27.72	4.32	5.55	1.62	4.99	0.78	0.29
65	62.55	5.84	11.05	2.72	29.21	4.84	5.85	1.63	5.00	0.83	0.28
65.5	52.62	4.38	9.68	4.26	24.57	3.63	5.12	2.55	4.80	0.71	0.50
66	59.55	4.88	10.48	2.80	27.81	4.05	5.54	1.68	5.02	0.73	0.30
66.5	47.82	3.33	8.15	3.13	22.33	2.76	4.31	1.88	5.18	0.64	0.44
67	58.66	4.67	10.11	2.72	27.39	3.87	5.35	1.63	5.12	0.72	0.31
67.5	60.37	5.37	11.26	3.02	28.19	4.46	5.96	1.81	4.73	0.75	0.30
68	60.36	3.93	10.76	4.23	28.19	3.27	5.69	2.54	4.95	0.57	0.45
68.5	58.20	4.60	9.89	2.99	27.18	3.82	5.23	1.79	5.19	0.73	0.34
69	58.31	4.62	10.63	3.18	27.23	3.83	5.62	1.91	4.84	0.68	0.34
69.5	59.53	4.25	10.11	4.07	27.80	3.52	5.35	2.44	5.20	0.66	0.46
70	62.10	5.07	10.75	2.59	29.00	4.21	5.68	1.56	5.10	0.74	0.27
70.5	60.79	4.85	12.12	2.90	28.39	4.02	6.41	1.74	4.43	0.63	0.27
71	56.51	5.44	13.40	2.49	26.39	4.51	7.09	1.49	3.72	0.64	0.21
71.5	58.38	5.41	10.55	2.44	27.26	4.49	5.58	1.47	4.89	0.80	0.26
72	58.22	4.34	10.20	2.89	27.19	3.60	5.40	1.73	5.04	0.67	0.32
72.5	56.27	4.36	9.99	2.72	26.28	3.62	5.28	1.63	4.97	0.68	0.31
73	56.54	4.36	10.18	2.16	26.40	3.62	5.39	1.30	4.90	0.67	0.24
73.5	61.56	4.92	10.18	2.23	28.75	4.08	5.39	1.34	5.34	0.76	0.25
74	56.35	4.19	9.67	2.81	26.32	3.48	5.11	1.68	5.15	0.68	0.33
74.5	30.55	2.48	6.53	1.89	14.27	2.05	3.46	1.13	4.13	0.59	0.33
75	60.62	5.13	8.95	1.75	28.31	4.25	4.74	1.05	5.98	0.90	0.22
75.5	59.04	5.19	10.39	2.32	27.57	4.31	5.50	1.39	5.02	0.78	0.25
76	59.54	5.57	11.17	2.92	27.80	4.62	5.91	1.75	4.71	0.78	0.30
76.5	30.04	*(2)*	5.12	1.54	14.03		2.71	0.92	5.18		0.34
77	27.85	2.07	6.25	1.68	13.00	1.72	3.30	1.01	3.94	0.52	0.30
77.5	55.08	5.38	9.91	2.06	25.72	4.46	5.24	1.24	4.91	0.85	0.24
78	56.91	4.65	10.35	2.97	26.58	3.86	5.48	1.78	4.85	0.71	0.33
78.5	59.54	4.89	10.33	2.58	27.81	4.06	5.46	1.55	5.09	0.74	0.28
79	57.85	4.94	10.19	2.20	27.01	4.10	5.39	1.32	5.01	0.76	0.24
79.5	60.12	4.54	9.90	2.43	28.07	3.77	5.24	1.46	5.36	0.72	0.28
80	58.69	4.01	10.26	3.72	27.41	3.33	5.43	2.23	5.05	0.61	0.41
80.5	54.22	3.96	9.90	2.59	25.32	3.29	5.24	1.55	4.83	0.63	0.30
81	59.22	4.38	10.47	2.33	27.66	3.64	5.54	1.40	4.99	0.66	0.25
81.5	55.96	4.41	10.40	3.06	26.13	3.66	5.50	1.84	4.75	0.66	0.33
82	59.03	4.37	10.57	2.78	27.57	3.63	5.59	1.67	4.93	0.65	0.30
82.5	53.09	5.05	9.17	2.68	24.79	4.19	4.85	1.61	5.11	0.86	0.33
83	54.65	4.82	10.53	2.45	25.52	4.00	5.57	1.47	4.58	0.72	0.26
83.5	53.49	4.15	9.72	2.57	24.98	3.44	5.14	1.54	4.86	0.67	0.30

84	52.45	3.78	9.42	2.37	24.49	3.14	4.99	1.42	4.91	0.63	0.29
84.5	45.86	3.52	8.57	2.50	21.41	2.93	4.53	1.50	4.72	0.65	0.33
85	57.73	4.04	10.40	2.78	26.96	3.36	5.50	1.67	4.90	0.61	0.30
85.5	57.34	4.48	10.59	2.12	26.78	3.72	5.60	1.27	4.78	0.66	0.23
86	56.65	5.05	11.34	2.60	26.46	4.19	6.00	1.56	4.41	0.70	0.26
86.5	66.32	4.04	9.01	1.82	30.97	3.36	4.76	1.09	6.50	0.70	0.23
87	73.34	3.93	9.01	1.41	34.25	3.26	4.77	0.85	7.19	0.68	0.18
87.5	62.85	5.93	11.16	2.31	29.35	4.92	5.90	1.39	4.97	0.83	0.23
88	61.96	6.69	12.28	2.26	28.93	5.55	6.50	1.35	4.45	0.85	0.21
88.5	63.45	6.26	12.24	2.46	29.63	5.20	6.47	1.48	4.58	0.80	0.23
89	67.52	6.49	13.02	2.41	31.53	5.39	6.89	1.44	4.58	0.78	0.21
89.5	62.99	6.24	11.57	2.70	29.42	5.18	6.12	1.62	4.80	0.85	0.26
90	70.80	5.25	11.35	2.13	33.06	4.36	6.00	1.28	5.51	0.73	0.21
90.5	71.11	5.59	9.01	1.87	33.21	4.64	4.77	1.12	6.97	0.97	0.24
91	76.60	4.22	8.00	1.99	35.77	3.50	4.23	1.19	8.45	0.83	0.28
91.5	68.69	5.42	10.55	2.04	32.08	4.49	5.58	1.22	5.75	0.81	0.22
92	70.08	5.72	11.67	1.95	32.73	4.75	6.17	1.17	5.30	0.77	0.19
92.5	71.87	4.54	8.57	1.68	33.56	3.77	4.53	1.01	7.40	0.83	0.22
93	69.97	5.76	11.28	2.12	32.67	4.78	5.97	1.27	5.47	0.80	0.21
93.5	66.43	4.52	8.97	2.47	31.02	3.75	4.75	1.48	6.54	0.79	0.31
94	61.84	5.68	10.72	1.90	28.88	4.72	5.67	1.14	5.09	0.83	0.20
94.5	58.24	5.04	9.77	1.61	27.20	4.19	5.17	0.96	5.26	0.81	0.19
95	59.77	5.20	9.28	2.73	27.91	4.31	4.91	1.63	5.68	0.88	0.33
95.5	60.90	5.02	9.55	2.20	28.44	4.17	5.05	1.32	5.63	0.83	0.26
96	67.54	4.32	7.96	1.64	31.54	3.58	4.21	0.98	7.49	0.85	0.23
96.5	54.61	3.28	7.68	1.22	25.50	2.72	4.07	0.73	6.27	0.67	0.18
97	56.95	3.68	8.28	1.81	26.60	3.06	4.38	1.08	6.07	0.70	0.25
97.5	72.71	3.79	8.21	1.38	33.96	3.14	4.35	0.83	7.81	0.72	0.19
98	67.19	4.56	9.94	1.51	31.38	3.79	5.26	0.91	5.97	0.72	0.17
98.5	61.68	6.47	13.36	2.64	28.80	5.37	7.07	1.58	4.08	0.76	0.22
99	67.50	3.78	7.92	1.81	31.52	3.14	4.19	1.08	7.53	0.75	0.26
99.5	39.11	3.48	8.01	2.34	18.26	2.89	4.24	1.41	4.31	0.68	0.33
100	62.83	4.54	10.53	1.80	29.34	3.77	5.57	1.08	5.27	0.68	0.19
100.5	55.77	4.57	10.20	1.86	26.04	3.79	5.40	1.11	4.83	0.70	0.21
101	52.09	3.69	7.96	2.76	24.33	3.06	4.21	1.65	5.78	0.73	0.39
101.5	53.84	4.54	9.93	1.98	25.14	3.77	5.25	1.19	4.79	0.72	0.23
102	63.93	4.70	9.79	1.94	29.86	3.90	5.18	1.16	5.77	0.75	0.22
102.5	65.33	4.97	10.21	2.45	30.51	4.12	5.40	1.47	5.65	0.76	0.27
103	67.41	4.77	10.12	2.53	31.48	3.96	5.35	1.52	5.88	0.74	0.28
103.5	58.86	4.45	10.60	1.74	27.49	3.69	5.61	1.05	4.90	0.66	0.19

104	56.70	4.67	10.56	1.80	26.48	3.88	5.59	1.08	4.74	0.69	0.19
104.5	56.68	4.51	13.27	2.31	26.47	3.74	7.02	1.38	3.77	0.53	0.20
105	57.44	4.23	10.55	2.00	26.83	3.51	5.58	1.20	4.81	0.63	0.22
105.5	60.41	4.22	10.48	1.74	28.21	3.50	5.54	1.04	5.09	0.63	0.19
106	51.73	4.54	9.68	1.64	24.16	3.77	5.12	0.98	4.72	0.74	0.19
106.5	27.34	*(0.7)*	*(4)*	0.64	12.77			0.38			
107	61.65	4.95	11.55	2.51	28.79	4.11	6.11	1.50	4.71	0.67	0.25
107.5	53.92	4.90	12.08	3.12	25.18	4.07	6.39	1.87	3.94	0.64	0.29
108	61.14	4.73	13.18	2.75	28.55	3.93	6.97	1.65	4.10	0.56	0.24
108.5	57.05	4.85	13.62	3.10	26.64	4.03	7.20	1.86	3.70	0.56	0.26
109	63.04	4.85	13.93	2.88	29.44	4.03	7.37	1.73	3.99	0.55	0.23
109.5	58.95	4.59	13.33	2.69	27.53	3.81	7.05	1.61	3.90	0.54	0.23
110	55.38	5.35	11.38	2.51	25.86	4.44	6.02	1.50	4.30	0.74	0.25
110.5	58.83	5.02	13.73	3.14	27.47	4.17	7.26	1.88	3.78	0.57	0.26
111	CORE LOSS										
111.5	59.94	5.05	13.47	2.86	27.99	4.19	7.13	1.71	3.93	0.59	0.24
112	54.62	4.73	11.42	3.17	25.51	3.92	6.04	1.90	4.22	0.65	0.31
112.5	53.86	4.93	11.52	2.98	25.15	4.10	6.09	1.79	4.13	0.67	0.29
113	59.81	4.82	12.42	2.73	27.93	4.00	6.57	1.63	4.25	0.61	0.25
113.5	63.47	5.18	13.92	2.71	29.64	4.30	7.36	1.63	4.03	0.58	0.22
114	60.71	5.07	14.77	2.87	28.35	4.20	7.81	1.72	3.63	0.54	0.22
114.5	59.77	4.99	13.76	2.69	27.91	4.15	7.28	1.61	3.83	0.57	0.22
115	59.31	4.50	13.64	2.76	27.70	3.73	7.21	1.65	3.84	0.52	0.23
115.5	57.99	4.23	13.98	2.51	27.08	3.51	7.39	1.51	3.66	0.48	0.20
116	60.48	4.62	12.76	2.63	28.24	3.84	6.75	1.57	4.18	0.57	0.23
116.5	59.45	4.99	11.55	2.45	27.76	4.14	6.11	1.47	4.54	0.68	0.24
117	25.04	*(0.7)*	5.93	0.62	11.69		3.14	0.37	3.73		0.12
117.5	*(8)*	*(0.5)*	*(3)*	*(0.2)*							
118	58.70	4.90	16.09	2.81	27.42	4.06	8.51	1.68	3.22	0.48	0.20
118.5	55.76	4.83	14.66	2.86	26.04	4.01	7.76	1.71	3.36	0.52	0.22
119	62.24	5.14	12.93	2.61	29.06	4.26	6.84	1.56	4.25	0.62	0.23
119.5	56.08	5.29	13.37	2.63	26.19	4.39	7.07	1.57	3.70	0.62	0.22
120	59.35	6.03	14.88	2.68	27.72	5.01	7.87	1.60	3.52	0.64	0.20
120.5	52.76	5.42	12.43	2.65	24.64	4.50	6.57	1.59	3.75	0.68	0.24
121	60.27	5.31	12.67	2.58	28.14	4.41	6.70	1.55	4.20	0.66	0.23
121.5	59.60	5.18	12.42	2.58	27.83	4.30	6.57	1.54	4.24	0.65	0.24
122	62.93	5.32	12.75	2.39	29.39	4.42	6.74	1.43	4.36	0.66	0.21
122.5	60.55	4.72	12.38	2.67	28.28	3.91	6.55	1.60	4.32	0.60	0.24
123	56.56	4.36	8.98	2.04	26.41	3.62	4.75	1.22	5.56	0.76	0.26
123.5	57.15	3.87	12.69	3.43	26.69	3.21	6.71	2.05	3.98	0.48	0.31

124	61.59	3.98	12.00	3.02	28.76	3.30	6.35	1.81	4.53	0.52	0.29
124.5	61.66	4.44	12.39	2.33	28.79	3.69	6.55	1.39	4.39	0.56	0.21
125	60.93	3.96	11.82	2.69	28.45	3.29	6.25	1.61	4.55	0.53	0.26
125.5	59.65	4.20	12.10	2.18	27.86	3.48	6.40	1.30	4.35	0.54	0.20
126	61.69	3.98	11.61	1.98	28.81	3.31	6.14	1.19	4.69	0.54	0.19
126.5	60.18	3.69	11.33	2.37	28.10	3.06	5.99	1.42	4.69	0.51	0.24
127	63.16	3.64	11.80	2.14	29.50	3.02	6.24	1.28	4.72	0.48	0.21
127.5	60.35	4.01	12.64	2.33	28.18	3.33	6.69	1.40	4.22	0.50	0.21
128	62.69	3.67	12.14	2.48	29.28	3.04	6.42	1.49	4.56	0.47	0.23
128.5	56.43	2.72	10.28	2.06	26.35	2.25	5.44	1.24	4.85	0.41	0.23
129	29.76	*(1.2)*	5.30	1.69	13.90		2.81	1.01	4.95		0.36
129.5	40.23	*(0.3)*	7.90	0.93	18.79		4.18	0.56	4.50		0.13
130	60.34	3.12	11.15	2.56	28.18	2.59	5.90	1.53	4.78	0.44	0.26
130.5	57.28	3.61	10.28	2.06	26.75	3.00	5.44	1.24	4.92	0.55	0.23
131	62.04	3.46	10.59	2.23	28.97	2.87	5.60	1.34	5.17	0.51	0.24
131.5	61.67	3.47	11.26	2.66	28.80	2.88	5.96	1.59	4.84	0.48	0.27
132	63.52	3.62	12.33	2.42	29.66	3.01	6.52	1.45	4.55	0.46	0.22
132.5	62.30	3.65	11.33	2.77	29.10	3.03	5.99	1.66	4.85	0.50	0.28
133	65.26	4.14	11.24	2.39	30.47	3.44	5.95	1.43	5.12	0.58	0.24
133.5	62.20	4.28	12.04	2.34	29.05	3.55	6.37	1.40	4.56	0.56	0.22
134	58.36	3.95	10.72	2.30	27.26	3.28	5.67	1.38	4.81	0.58	0.24
134.5	55.89	4.50	13.17	2.40	26.10	3.74	6.97	1.44	3.74	0.54	0.21
135	20.33	*(0.4)*	*(4)*	0.99	9.49			0.60			
135.5	60.23	5.87	13.82	2.86	28.13	4.87	7.31	1.71	3.85	0.67	0.23
136	57.69	4.72	13.86	3.05	26.94	3.92	7.33	1.83	3.68	0.53	0.25
136.5	57.90	4.89	14.53	3.01	27.04	4.06	7.69	1.81	3.52	0.53	0.23
137	60.02	5.12	14.88	3.18	28.03	4.25	7.87	1.90	3.56	0.54	0.24
137.5	61.71	5.20	12.01	2.37	28.82	4.32	6.35	1.42	4.54	0.68	0.22
138	60.43	5.22	13.92	2.97	28.22	4.33	7.36	1.78	3.83	0.59	0.24
138.5	59.74	5.69	14.54	3.17	27.90	4.73	7.69	1.90	3.63	0.61	0.25
139	60.29	5.31	14.22	3.17	28.16	4.41	7.52	1.90	3.74	0.59	0.25
139.5	58.91	4.91	12.83	3.14	27.51	4.07	6.79	1.88	4.05	0.60	0.28
140	60.97	5.23	12.68	2.87	28.47	4.34	6.71	1.72	4.25	0.65	0.26
140.5	64.08	4.89	12.10	2.54	29.93	4.06	6.40	1.52	4.68	0.63	0.24
141	61.53	5.06	14.52	3.02	28.74	4.20	7.68	1.81	3.74	0.55	0.24
141.5	57.71	4.16	13.60	3.87	26.95	3.46	7.20	2.32	3.75	0.48	0.32
142	66.96	4.20	12.63	2.67	31.27	3.49	6.68	1.60	4.68	0.52	0.24
142.5	64.28	3.42	11.48	6.28	30.02	2.84	6.07	3.76	4.94	0.47	0.62
143	63.15	3.30	10.45	3.30	29.49	2.74	5.53	1.98	5.33	0.50	0.36
143.5	67.34	4.02	11.69	2.80	31.45	3.34	6.18	1.68	5.09	0.54	0.27

144	57.34	4.11	12.82	3.63	26.78	3.41	6.78	2.17	3.95	0.50	0.32
144.5	25.74	*(1.2)*	5.08	1.89	12.02		2.69	1.13	4.47		0.42
145	57.35	3.55	11.63	2.90	26.78	2.94	6.15	1.74	4.36	0.48	0.28
145.5	23.92	*(1.4)*	5.15	1.45	11.17		2.73	0.87	4.10		0.32
146	24.88	*(0)*	*(1)*	*(0)*	11.62						
146.5	29.41	*(1.6)*	5.92	1.50	13.73		3.13	0.90	4.39		0.29
147	62.20	4.65	13.98	3.56	29.05	3.86	7.40	2.14	3.93	0.52	0.29
147.5	66.97	3.24	10.62	5.78	31.27	2.69	5.62	3.46	5.56	0.48	0.62
148	73.62	2.30	9.82	1.77	34.38	1.91	5.19	1.06	6.62	0.37	0.20
148.5	67.00	3.92	11.58	2.59	31.29	3.26	6.12	1.55	5.11	0.53	0.25
149	63.55	3.24	10.01	2.62	29.68	2.69	5.30	1.57	5.60	0.51	0.30
149.5	71.67	2.83	9.25	2.67	33.47	2.35	4.89	1.60	6.84	0.48	0.33
150	59.83	4.44	11.93	2.32	27.94	3.69	6.31	1.39	4.43	0.58	0.22
150.5	62.17	4.35	12.78	3.04	29.03	3.61	6.76	1.82	4.30	0.53	0.27
151	62.40	3.69	11.04	3.43	29.14	3.06	5.84	2.06	4.99	0.52	0.35
151.5	61.00	4.81	14.13	2.29	28.49	4.00	7.47	1.37	3.81	0.53	0.18
152	64.34	4.70	12.64	2.18	30.05	3.90	6.69	1.30	4.49	0.58	0.20
152.5	61.15	4.86	14.02	2.38	28.56	4.03	7.42	1.43	3.85	0.54	0.19
153	62.08	4.52	14.53	2.54	28.99	3.75	7.68	1.52	3.77	0.49	0.20
153.5	62.58	4.53	14.78	2.47	29.22	3.76	7.82	1.48	3.74	0.48	0.19
154	64.36	3.26	10.74	2.78	30.06	2.71	5.68	1.66	5.29	0.48	0.29
154.5	53.75	3.54	15.16	2.95	25.10	2.94	8.02	1.77	3.13	0.37	0.22
155	52.17	3.80	14.60	3.11	24.36	3.16	7.72	1.87	3.15	0.41	0.24
155.5	63.64	5.79	12.48	2.57	29.72	4.81	6.60	1.54	4.50	0.73	0.23
156	65.36	4.41	13.85	3.13	30.52	3.66	7.33	1.88	4.16	0.50	0.26
156.5	61.58	4.30	11.03	2.23	28.76	3.57	5.83	1.33	4.93	0.61	0.23
157	60.02	4.41	13.13	3.27	28.03	3.66	6.94	1.96	4.04	0.53	0.28
157.5	59.20	4.21	11.66	2.38	27.65	3.49	6.17	1.43	4.48	0.57	0.23
158	63.95	4.19	11.75	2.56	29.86	3.48	6.22	1.54	4.80	0.56	0.25
158.5	58.77	4.42	12.37	2.65	27.45	3.67	6.54	1.59	4.19	0.56	0.24
159	57.69	4.27	12.57	2.62	26.94	3.54	6.65	1.57	4.05	0.53	0.24
159.5	60.78	4.41	12.42	2.00	28.38	3.66	6.57	1.20	4.32	0.56	0.18
160	62.36	3.89	12.02	2.42	29.12	3.23	6.36	1.45	4.58	0.51	0.23
160.5	63.37	4.14	13.33	2.70	29.59	3.44	7.05	1.62	4.20	0.49	0.23
161	65.01	3.58	11.03	2.71	30.36	2.97	5.84	1.63	5.20	0.51	0.28
161.5	60.84	3.80	11.96	3.65	28.41	3.15	6.33	2.19	4.49	0.50	0.35
162	60.66	4.19	12.91	3.27	28.33	3.48	6.83	1.96	4.15	0.51	0.29
162.5	61.50	4.40	13.11	2.51	28.72	3.65	6.94	1.50	4.14	0.53	0.22
163	25.04	*(1.2)*	8.47	1.29	11.69		4.48	0.77	2.61		0.17
163.5	59.99	3.81	12.23	2.47	28.01	3.16	6.47	1.48	4.33	0.49	0.23

164	62.33	3.78	12.54	2.57	29.11	3.13	6.63	1.54	4.39	0.47	0.23
164.5	61.20	3.49	12.36	2.95	28.58	2.90	6.54	1.77	4.37	0.44	0.27
165	61.60	3.79	13.50	2.62	28.77	3.15	7.14	1.57	4.03	0.44	0.22
165.5	53.34	4.07	12.63	2.85	24.91	3.38	6.68	1.71	3.73	0.51	0.26
166	59.24	3.75	12.52	2.60	27.67	3.11	6.63	1.56	4.18	0.47	0.24
166.5	28.06	*(0.9)*	8.32	1.12	13.10		4.40	0.67	2.98		0.15
167	12.24	*(0.3)*	*(3)*	*(0.2)*	5.71						
167.5	47.12	2.75	17.07	4.21	22.00	2.28	9.03	2.52	2.44	0.25	0.28
168	50.50	2.76	18.26	4.41	23.58	2.29	9.66	2.64	2.44	0.24	0.27
168.5	53.11	2.99	16.06	3.19	24.80	2.48	8.49	1.91	2.92	0.29	0.22
169	53.80	2.67	19.17	3.58	25.12	2.22	10.14	2.15	2.48	0.22	0.21
169.5	52.49	2.70	18.70	3.19	24.51	2.24	9.89	1.91	2.48	0.23	0.19
170	46.94	2.17	17.42	3.37	21.92	1.80	9.21	2.02	2.38	0.20	0.22
170.5	50.43	2.40	19.44	3.47	23.55	2.00	10.28	2.08	2.29	0.19	0.20
171	49.10	2.44	20.02	4.61	22.93	2.03	10.59	2.76	2.17	0.19	0.26
171.5	40.42	*(1.5)*	14.00	1.48	18.88		7.40	0.89	2.55		0.12
172	44.06	*(2)*	18.64	2.36	20.58		9.86	1.41	2.09		0.14
172.5	38.96	2.10	17.85	3.29	18.19	1.74	9.45	1.97	1.93	0.18	0.21
173	59.70	3.07	17.96	5.26	27.88	2.55	9.50	3.15	2.93	0.27	0.33
173.5	64.29	3.48	18.38	2.64	30.03	2.89	9.73	1.58	3.09	0.30	0.16
174	60.48	2.98	16.48	2.02	28.24	2.47	8.72	1.21	3.24	0.28	0.14
174.5	60.67	3.79	19.84	2.72	28.33	3.14	10.50	1.63	2.70	0.30	0.16
175	61.96	4.39	16.60	3.05	28.94	3.64	8.78	1.83	3.29	0.41	0.21
175.5	60.47	4.51	17.26	2.51	28.24	3.74	9.13	1.50	3.09	0.41	0.16
176	66.56	4.94	14.66	3.06	31.08	4.10	7.76	1.83	4.01	0.53	0.24
176.5	63.59	5.42	16.79	2.59	29.70	4.49	8.88	1.55	3.34	0.51	0.17
177	62.25	4.94	17.97	3.17	29.07	4.10	9.51	1.90	3.06	0.43	0.20
177.5	60.01	5.13	15.88	2.86	28.02	4.26	8.40	1.71	3.34	0.51	0.20
178	60.26	5.25	14.55	2.14	28.14	4.36	7.69	1.28	3.66	0.57	0.17
178.5	62.22	5.44	14.34	2.22	29.06	4.52	7.59	1.33	3.83	0.60	0.18
179	65.34	4.59	14.90	2.87	30.51	3.81	7.88	1.72	3.87	0.48	0.22
179.5	57.10	4.16	14.49	2.15	26.66	3.46	7.66	1.29	3.48	0.45	0.17
180	59.86	4.86	16.14	2.26	27.95	4.04	8.54	1.35	3.27	0.47	0.16
180.5	64.13	4.29	17.00	3.06	29.95	3.56	8.99	1.83	3.33	0.40	0.20
181	59.82	4.41	16.57	2.95	27.94	3.66	8.76	1.77	3.19	0.42	0.20
181.5	61.19	5.69	14.83	2.76	28.58	4.72	7.85	1.65	3.64	0.60	0.21
182	62.63	4.77	15.33	2.83	29.25	3.96	8.11	1.70	3.61	0.49	0.21
182.5	62.70	5.01	14.62	3.46	29.28	4.16	7.73	2.07	3.79	0.54	0.27
183	59.06	4.66	16.49	2.93	27.58	3.87	8.72	1.75	3.16	0.44	0.20
183.5	58.31	4.86	13.91	3.02	27.23	4.03	7.36	1.81	3.70	0.55	0.25

184	60.11	4.78	16.01	2.95	28.07	3.97	8.47	1.77	3.32	0.47	0.21
184.5	39.43	*(1.8)*	9.88	2.20	18.41		5.23	1.32	3.52		0.25
185	37.27	*(1.7)*	8.91	2.50	17.40		4.71	1.50	3.69		0.32
185.5	43.78	*(1.8)*	9.58	2.17	20.45		5.07	1.30	4.03		0.26
186	58.78	4.00	14.93	3.49	27.45	3.32	7.90	2.09	3.48	0.42	0.27
186.5	53.41	2.88	19.63	3.32	24.94	2.39	10.39	1.99	2.40	0.23	0.19
187	55.31	*(1.9)*	20.14	2.34	25.83		10.65	1.40	2.43		0.13
187.5	36.98	*(0.8)*	17.93	2.90	17.27		9.48	1.74	1.82		0.18
188	36.96	*(1.4)*	12.27	3.52	17.26		6.49	2.11	2.66		0.32
188.5	39.57	2.91	15.87	4.79	18.48	2.41	8.40	2.87	2.20	0.29	0.34
189	31.98	2.12	13.95	4.15	14.93	1.76	7.38	2.48	2.02	0.24	0.34
189.5	56.80	*(1.3)*	8.61	2.55	26.53		4.56	1.53	5.82		0.34
190	44.10	3.02	20.00	4.35	20.59	2.51	10.58	2.61	1.95	0.24	0.25
191	CORE LOSS										
192	56.30	3.30	16.73	3.79	26.29	2.74	8.85	2.27	2.97	0.31	0.26
192.5	53.64	3.69	15.55	3.51	25.05	3.06	8.22	2.10	3.05	0.37	0.26
193	56.26	4.95	15.56	3.13	26.27	4.11	8.23	1.88	3.19	0.50	0.23
193.5	62.60	5.16	16.56	3.09	29.24	4.28	8.76	1.85	3.34	0.49	0.21
194	68.23	5.15	15.32	4.28	31.87	4.27	8.11	2.56	3.93	0.53	0.32
194.5	CORE LOSS										
195	56.98	4.45	14.65	2.59	26.61	3.70	7.75	1.55	3.43	0.48	0.20
195.5	64.31	4.53	13.58	2.39	30.03	3.76	7.19	1.44	4.18	0.52	0.20
196	63.61	5.21	14.18	2.30	29.71	4.32	7.50	1.38	3.96	0.58	0.18
196.5	64.02	4.57	13.40	2.47	29.90	3.80	7.09	1.48	4.22	0.54	0.21
197	68.61	3.69	12.78	2.37	32.04	3.06	6.76	1.42	4.74	0.45	0.21
197.5	61.62	3.41	12.75	2.17	28.77	2.83	6.74	1.30	4.27	0.42	0.19
198	62.76	3.27	12.28	2.14	29.31	2.71	6.50	1.28	4.51	0.42	0.20
198.5	60.68	5.32	15.51	2.83	28.34	4.42	8.21	1.69	3.45	0.54	0.21
199	62.60	4.66	13.63	2.49	29.23	3.87	7.21	1.49	4.05	0.54	0.21
199.5	53.40	3.95	14.80	2.64	24.94	3.28	7.83	1.58	3.19	0.42	0.20
200	52.94	5.44	17.86	3.91	24.72	4.52	9.45	2.34	2.62	0.48	0.25
200.5	60.63	5.37	15.74	2.47	28.31	4.46	8.32	1.48	3.40	0.54	0.18
201	52.87	7.42	18.43	4.21	24.69	6.16	9.75	2.52	2.53	0.63	0.26
201.5	55.19	6.67	17.19	2.41	25.78	5.54	9.10	1.44	2.83	0.61	0.16
202	57.37	5.22	15.31	3.18	26.79	4.33	8.10	1.91	3.31	0.53	0.24
202.5	62.32	4.61	14.20	4.51	29.10	3.83	7.51	2.70	3.87	0.51	0.36
203	72.37	2.79	12.41	1.73	33.80	2.32	6.56	1.04	5.15	0.35	0.16
203.5	54.85	4.92	15.29	2.98	25.61	4.08	8.09	1.78	3.17	0.50	0.22
204	62.40	2.21	17.68	3.93	29.14	1.83	9.35	2.35	3.12	0.20	0.25
204.5	35.57	*(1.2)*	12.73	3.86	16.61		6.73	2.31	2.47		0.34

205	58.96	4.60	15.41	2.91	27.53	3.82	8.15	1.74	3.38	0.47	0.21
205.5	34.96	*(1.8)*	8.46	1.79	16.33		4.48	1.07	3.65		0.24
206	58.81	5.20	15.34	2.83	27.47	4.32	8.11	1.69	3.39	0.53	0.21
206.5	60.41	4.66	14.72	2.93	28.21	3.87	7.79	1.76	3.62	0.50	0.23
207	61.00	3.92	15.68	3.26	28.49	3.25	8.30	1.95	3.43	0.39	0.24
207.5	71.44	*(0.5)*	7.42	4.15	33.36		3.92	2.49	8.50		0.63
208	59.66	*(1.7)*	17.02	4.54	27.86		9.00	2.72	3.09		0.30
208.5	46.19	*(0.2)*	22.89	3.36	21.57		12.11	2.01	1.78		0.17
209	55.98	*(1.2)*	22.04	3.27	26.14		11.66	1.96	2.24		0.17
209.5	72.87	*(0)*	9.12	3.23	34.03		4.83	1.94	7.05		0.40
210	70.28	*(0)*	8.12	1.02	32.82		4.30	0.61	7.64		0.14
210.5	59.22	*(0.5)*	14.58	3.52	27.66		7.71	2.11	3.59		0.27
211	62.42	*(0.1)*	16.00	2.73	29.15		8.47	1.64	3.44		0.19
211.5	66.93	*(0)*	13.35	3.31	31.26		7.06	1.98	4.43		0.28
212	61.39	*(0.6)*	16.49	2.01	28.67		8.72	1.20	3.29		0.14
212.5	55.45	*(0)*	22.55	2.76	25.89		11.93	1.65	2.17		0.14
213	75.75	*(0.5)*	5.03	1.35	35.37		2.66	0.81	13.30		0.30
213.5	67.19	*(0.7)*	8.31	5.54	31.38		4.40	3.32	7.14		0.76
214	58.47	*(1.4)*	20.11	3.70	27.31		10.64	2.22	2.57		0.21
214.5	76.14	*(0)*	5.94	1.16	35.56		3.14	0.69	11.31		0.22
215	74.13	*(0.4)*	10.41	0.65	34.62		5.51	0.39	6.29		0.07
215.5	77.34	*(0.1)*	5.29	0.62	36.12		2.80	0.37	12.91		0.13
216	78.57	*(0.2)*	8.50	0.47	36.69		4.50	0.28	8.16		0.06
216.5	84.58	*(0)*	5.60	1.78	39.50		2.96	1.07	13.33		0.36
217	83.11	*(0)*	*(4)*	0.26	38.81			0.16			
217.5	54.77	3.47	19.88	2.73	25.58	2.88	10.51	1.64	2.43	0.27	0.16
218	52.12	2.96	12.48	2.05	24.34	2.46	6.60	1.23	3.69	0.37	0.19
218.5	57.05	2.89	10.80	1.79	26.64	2.40	5.71	1.07	4.66	0.42	0.19
219	55.97	3.20	12.53	1.97	26.14	2.66	6.63	1.18	3.94	0.40	0.18
219.5	46.14	*(1.6)*	7.20	1.41	21.55		3.81	0.84	5.66		0.22
220	63.78	2.64	10.63	1.86	29.79	2.19	5.62	1.12	5.30	0.39	0.20
220.5	56.14	*(2)*	8.28	1.58	26.22		4.38	0.94	5.99		0.22
221	57.65	*(1.8)*	8.27	1.45	26.92		4.38	0.87	6.15		0.20
221.5	66.38	2.70	11.34	1.90	31.00	2.24	6.00	1.14	5.17	0.37	0.19
222	59.61	2.39	10.17	1.56	27.84	1.98	5.38	0.94	5.17	0.37	0.17
222.5	60.24	2.39	11.07	1.80	28.13	1.99	5.85	1.08	4.81	0.34	0.18
223	76.45	*(0.7)*	5.17	1.19	35.70		2.73	0.71	13.06		0.26
223.5	68.14	*(0.4)*	*(5)*	1.15	31.82			0.69			
224	67.96	2.39	8.33	1.64	31.74	1.98	4.40	0.98	7.21	0.45	0.22
224.5	75.00	2.26	6.91	1.41	35.02	1.88	3.66	0.84	9.58	0.51	0.23

225	71.79	2.31	9.20	1.68	33.53	1.92	4.87	1.01	6.89	0.39	0.21
225.5	57.01	2.48	8.15	1.57	26.62	2.06	4.31	0.94	6.18	0.48	0.22
226	43.95	*(1.3)*	7.45	1.98	20.53		3.94	1.19	5.21		0.30
226.5	59.03	4.09	11.53	2.19	27.57	3.40	6.10	1.31	4.52	0.56	0.22
227	64.60	2.47	7.88	1.49	30.17	2.05	4.17	0.89	7.24	0.49	0.21
227.5	60.84	3.47	10.45	2.08	28.41	2.88	5.53	1.25	5.14	0.52	0.23
228	72.51	2.17	6.20	1.45	33.86	1.80	3.28	0.87	10.32	0.55	0.26
228.5	49.58	2.21	6.09	1.40	23.15	1.83	3.22	0.84	7.19	0.57	0.26
229	56.47	3.55	9.46	1.69	26.37	2.95	5.00	1.01	5.27	0.59	0.20
229.5	63.17	3.39	8.57	1.66	29.50	2.81	4.53	0.99	6.51	0.62	0.22
230	50.75	3.53	7.63	1.70	23.70	2.93	4.04	1.02	5.87	0.73	0.25
230.5	59.34	3.76	8.56	1.74	27.71	3.12	4.53	1.05	6.12	0.69	0.23
231	43.15	3.31	6.96	2.01	20.15	2.74	3.68	1.21	5.47	0.75	0.33
231.5	70.64	2.80	8.67	1.50	32.99	2.32	4.59	0.90	7.19	0.51	0.20
232	44.99	4.03	8.61	2.08	21.01	3.35	4.56	1.25	4.61	0.73	0.27
232.5	60.81	3.05	9.14	1.38	28.40	2.53	4.84	0.83	5.87	0.52	0.17
233	58.66	2.77	6.57	1.23	27.40	2.30	3.48	0.74	7.88	0.66	0.21
233.5	56.30	2.93	6.10	1.32	26.29	2.43	3.23	0.79	8.15	0.75	0.24
234	66.14	4.01	8.76	1.84	30.89	3.33	4.63	1.10	6.66	0.72	0.24
234.5	59.97	4.05	9.04	1.86	28.01	3.36	4.78	1.11	5.86	0.70	0.23
235	56.87	3.43	7.85	1.49	26.56	2.84	4.15	0.89	6.39	0.68	0.22
235.5	64.78	3.23	7.75	1.50	30.25	2.68	4.10	0.90	7.38	0.65	0.22
236	65.44	3.79	8.86	1.64	30.56	3.15	4.69	0.98	6.52	0.67	0.21
236.5	51.81	3.70	7.28	1.39	24.19	3.07	3.85	0.84	6.28	0.80	0.22
237	52.42	4.30	9.26	1.79	24.48	3.57	4.90	1.07	5.00	0.73	0.22
237.5	64.77	3.48	7.83	1.52	30.25	2.89	4.14	0.91	7.30	0.70	0.22
238	68.33	3.65	8.62	1.50	31.91	3.03	4.56	0.90	7.00	0.67	0.20
238.5	47.30	3.28	6.82	1.62	22.09	2.72	3.61	0.97	6.13	0.75	0.27
239	62.80	3.47	8.28	1.66	29.33	2.88	4.38	0.99	6.70	0.66	0.23
239.5	52.75	3.30	7.84	1.68	24.64	2.74	4.15	1.01	5.94	0.66	0.24
240	71.11	3.36	8.68	1.66	33.21	2.79	4.59	1.00	7.23	0.61	0.22
240.5	69.93	3.49	8.64	1.64	32.66	2.90	4.57	0.98	7.15	0.63	0.22
241	65.34	3.35	7.67	1.39	30.51	2.78	4.06	0.83	7.52	0.69	0.21
241.5	56.58	3.65	9.20	1.73	26.42	3.03	4.86	1.04	5.43	0.62	0.21
242	45.40	2.20	6.62	2.45	21.20	1.83	3.50	1.47	6.05	0.52	0.42
242.5	57.73	3.49	9.87	1.56	26.96	2.90	5.22	0.93	5.17	0.56	0.18
243	39.62	*(1.5)*	5.52	1.34	18.50		2.92	0.80	6.33		0.27
243.5	66.96	2.95	7.95	1.42	31.27	2.45	4.21	0.85	7.43	0.58	0.20
244	45.63	2.58	6.84	2.10	21.31	2.14	3.62	1.26	5.89	0.59	0.35
244.5	68.03	3.51	7.77	1.06	31.77	2.91	4.11	0.64	7.72	0.71	0.15

245	65.17	3.57	8.59	1.49	30.43	2.96	4.54	0.89	6.70	0.65	0.20
245.5	51.85	3.81	8.57	1.85	24.21	3.17	4.53	1.11	5.34	0.70	0.24
246	58.69	3.77	9.07	1.63	27.41	3.13	4.80	0.98	5.71	0.65	0.20
246.5	49.78	3.23	7.07	1.58	23.25	2.68	3.74	0.95	6.21	0.72	0.25
247	64.21	3.05	5.95	1.11	29.98	2.54	3.15	0.67	9.53	0.81	0.21
247.5	55.49	3.04	6.32	1.11	25.92	2.52	3.34	0.67	7.75	0.75	0.20
248	56.51	2.91	6.90	1.07	26.39	2.42	3.65	0.64	7.23	0.66	0.18
248.5	36.57	2.88	5.03	1.48	17.08	2.39	2.66	0.89	6.42	0.90	0.33
249	51.12	4.01	7.67	1.84	23.88	3.33	4.05	1.10	5.89	0.82	0.27
249.5	60.25	3.62	6.70	1.05	28.14	3.01	3.55	0.63	7.93	0.85	0.18
250	56.56	4.60	8.94	1.83	26.42	3.82	4.73	1.10	5.59	0.81	0.23
250.5	51.67	4.11	7.37	1.62	24.13	3.41	3.90	0.97	6.19	0.88	0.25
251	48.64	4.49	8.22	1.67	22.71	3.73	4.35	1.00	5.22	0.86	0.23
251.5	44.44	4.23	7.26	1.75	20.75	3.51	3.84	1.05	5.40	0.91	0.27
252	57.37	4.06	7.76	1.54	26.79	3.37	4.11	0.92	6.52	0.82	0.22
252.5	63.99	5.18	10.35	1.56	29.88	4.30	5.47	0.93	5.46	0.79	0.17
253	60.47	3.71	8.19	1.72	28.24	3.08	4.33	1.03	6.52	0.71	0.24
253.5	44.47	3.28	5.42	1.11	20.77	2.72	2.87	0.67	7.24	0.95	0.23
254	75.31	4.46	7.05	1.04	35.17	3.70	3.73	0.62	9.43	0.99	0.17
254.5	80.07	4.39	6.05	0.81	37.39	3.64	3.20	0.49	11.68	1.14	0.15
255	66.15	4.77	7.80	1.36	30.89	3.96	4.13	0.82	7.49	0.96	0.20
255.5	65.87	5.06	8.99	1.43	30.76	4.20	4.76	0.86	6.47	0.88	0.18
256	60.64	5.52	10.07	1.90	28.32	4.58	5.33	1.14	5.31	0.86	0.21
256.5	55.52	4.79	8.15	1.59	25.93	3.98	4.31	0.95	6.01	0.92	0.22
257	50.62	6.00	7.91	1.99	23.64	4.98	4.18	1.19	5.65	1.19	0.28
257.5	50.58	5.24	8.73	1.88	23.62	4.35	4.62	1.13	5.11	0.94	0.24
258	69.72	5.39	9.18	1.12	32.56	4.47	4.86	0.67	6.70	0.92	0.14
258.5	65.86	4.33	7.46	0.89	30.76	3.60	3.95	0.53	7.80	0.91	0.13
259	68.96	4.46	8.52	1.33	32.20	3.70	4.51	0.80	7.14	0.82	0.18
259.5	69.33	4.10	8.28	1.42	32.38	3.40	4.38	0.85	7.39	0.78	0.19
260	76.68	2.75	6.66	1.18	35.81	2.28	3.52	0.71	10.16	0.65	0.20
260.5	76.12	2.80	6.17	1.15	35.55	2.32	3.26	0.69	10.90	0.71	0.21
261	74.52	3.49	8.64	1.31	34.80	2.90	4.57	0.78	7.61	0.63	0.17
261.5	72.04	2.42	6.94	1.15	33.64	2.01	3.67	0.69	9.17	0.55	0.19
262	71.30	2.07	6.35	1.23	33.29	1.72	3.36	0.74	9.91	0.51	0.22
262.5	71.31	3.60	8.48	1.57	33.30	2.99	4.48	0.94	7.43	0.67	0.21
263	69.00	3.20	7.82	1.44	32.22	2.66	4.14	0.87	7.79	0.64	0.21
263.5	73.15	2.40	5.88	1.20	34.16	1.99	3.11	0.72	10.99	0.64	0.23
264	70.07	2.94	7.13	1.34	32.72	2.44	3.77	0.81	8.68	0.65	0.21
264.5	59.84	3.99	7.31	1.31	27.95	3.31	3.87	0.79	7.22	0.86	0.20

265	92.78	2.38	(4)	0.99	43.33	1.98		0.59			
265.5	80.47	2.79	(4)	0.68	37.58	2.32		0.41			
266	67.26	2.87	7.41	1.43	31.41	2.38	3.92	0.85	8.02	0.61	0.22
266.5	68.83	9.99	9.48	0.36	32.14	8.29	5.01	0.22	6.41	1.65	0.04
267	42.79	2.02	(3)	1.37	19.98	1.67		0.82			
267.5	65.73	(2)	(4)	0.77	30.70			0.46			
268	53.75	3.57	13.90	1.83	25.10	2.96	7.36	1.09	3.41	0.40	0.15
268.5	26.70	(1.2)	5.58	0.84	12.47		2.95	0.51	4.22		0.17
269	15.93	(0.5)	(2)	0.37	7.44			0.22			
269.5	47.71	2.49	5.26	1.71	22.28	2.07	2.78	1.03	8.00	0.74	0.37
270	45.40	(1.9)	(4)	0.89	21.20			0.54			
270.5	40.62	(1.5)	(4)	1.23	18.97			0.74			
271	23.92	(0.8)	(3)	0.70	11.17			0.42			
271.5	11.34	(0.1)	(1)	0.39	5.29			0.23			
272	27.08	(0.7)	(3)	0.78	12.65			0.46			
272.5	20.26	(0.5)	(2)	0.39	9.46			0.24			
273	38.85	(1.2)	(3)	1.05	18.14			0.63			
273.5	60.14	2.58	(5)	1.33	28.08	2.14		0.80			
274	60.32	2.66	5.77	1.20	28.17	2.21	3.05	0.72	9.23	0.72	0.24
274.5	26.26	(1)	(3)	0.85	12.26			0.51			
275	66.40	4.12	8.88	1.47	31.01	3.42	4.70	0.88	6.60	0.73	0.19
275.5	40.51	(1.3)	(3)	0.90	18.92			0.54			
276	21.08	(0.6)	(3)	0.61	9.85			0.37			
276.5	61.49	5.92	11.81	2.11	28.72	4.92	6.25	1.26	4.60	0.79	0.20
277	48.90	(1.9)	(4)	1.29	22.84			0.78			
277.5	63.81	3.93	7.85	1.85	29.80	3.26	4.16	1.11	7.17	0.79	0.27
278	62.63	3.60	7.01	1.53	29.25	2.99	3.71	0.92	7.89	0.81	0.25
278.5	55.82	4.83	9.79	1.86	26.07	4.01	5.18	1.12	5.03	0.77	0.22
279	12.10	(0.1)	(1)	0.33	5.65			0.20			
279.5	56.05	(1.6)	(5)	0.77	26.17			0.46			
280	42.95	(1.2)	(3)	0.58	20.06			0.35			
280.5	56.28	4.69	8.88	1.84	26.28	3.89	4.70	1.10	5.59	0.83	0.23
281	54.39	2.97	6.67	1.54	25.40	2.47	3.53	0.92	7.20	0.70	0.26
281.5	37.94	(1.8)	(4)	1.35	17.72			0.81			
282	35.77	(1.3)	5.51	1.09	16.71		2.91	0.66	5.73		0.22
282.5	17.67	(0.7)	(2)	0.43	8.25			0.26			
283	25.29	(0.2)	(1)	0.46	11.81			0.27			
283.5	20.36	(0)	(1)	(0.2)	9.51						
284	9.04	(0.1)	(1)	0.24	4.22			0.14			
284.5	15.11	(0.3)	(1)	0.47	7.06			0.28			

285	20.98	*(0)*	*(1)*	0.31	9.80			0.18			
285.5	33.19	*(0)*	*(0)*	*(0.2)*	15.50						
286	29.75	*(0)*	*(0)*	*(0.2)*	13.89						
286.5	12.46	*(0)*	*(1)*	0.24	5.82			0.15			
287	19.91	*(0)*	*(1)*	0.27	9.30			0.16			
287.5	*(5)*	*(0)*	*(1)*	0.25				0.15			
288	35.68	*(0)*	*(0)*	*(0.2)*	16.66						
288.5	22.47	*(0)*	*(0)*	0.24	10.49			0.15			
289	16.19	*(0)*	*(0)*	0.24	7.56			0.14			
289.5	24.00	*(0)*	*(1)*	0.32	11.21			0.19			
290	25.58	*(0.1)*	*(1)*	0.40	11.95			0.24			
290.5	47.62	*(0.1)*	*(1)*	0.41	22.24			0.24			
291	*(8)*	*(0)*	*(1)*	0.28				0.17			
291.5	55.21	4.40	8.66	2.78	25.78	3.65	4.58	1.67	5.63	0.80	0.36
292	12.84	*(0)*	*(1)*	0.29	5.99			0.17			
292.5	26.42	*(0.2)*	*(2)*	0.54	12.34			0.32			
293	22.48	*(0)*	*(1)*	0.32	10.50			0.19			
293.5	30.81	*(0)*	*(1)*	0.43	14.39			0.26			
294	9.19	*(0)*	*(0)*	0.27	4.29			0.16			
294.5	54.12	3.29	7.06	1.77	25.27	2.73	3.73	1.06	6.77	0.73	0.28
295	27.85	*(0.1)*	*(1)*	0.43	13.01			0.26			
295.5	*(8)*	*(0)*	*(0)*	*(0.2)*							
296	47.11	*(1)*	5.51	0.88	22.00		2.92	0.53	7.55		0.18
296.5	58.74	*(1.5)*	5.38	0.91	27.43		2.84	0.54	9.65		0.19
297	37.73	*(0.8)*	*(4)*	0.77	17.62			0.46			
297.5	36.35	*(0.6)*	*(4)*	0.81	16.97			0.49			
298	12.91	*(0.1)*	*(1)*	0.37	6.03			0.22			
298.5	38.57	*(0.5)*	*(3)*	0.68	18.01			0.41			
299	29.50	*(0.4)*	*(4)*	0.74	13.77			0.44			
299.5	43.06	*(0.6)*	*(3)*	0.81	20.11			0.49			
300	17.28	*(0)*	*(1)*	0.34	8.07			0.21			

Correlation coefficient (r) of Si and Al from Triassic section (~0 - 217 m) = 0.012
Correlation coefficient (r) of Si and Al from Permian section (~217 - 300 m) = 0.094

Table SI-3 Geochemical data of Carbon and Nitrogen from Bicheno-5 core, Tasmania.

Sample Number	Depth Start (m)	Depth end (m)	Sample Depth	Nitrogen (%)	Carbon (%)	d^{13}Corg [VPDB] (‰)	C/N
S1	7	7.2	7.1	0.09	1.69	-24.78	18.45
S4	10	10.2	10.1	0.02	0.36	-25.65	18.35
S5	10.5	10.7	10.6	0.05	3.17	-25.51	61.37
S7	12	12.3	12.15	0.13	0.21	-25.95	1.62
S9	14.3	14.6	14.45	0.04	0.07	-26.83	1.64
S13	17	17.2	17.1	0.01	0.04	-26.33	3.51
S15	18	18.4	18.2	0.22	0.18	-26.42	0.81
S17	19	19.3	19.15	0.02	0.12	-25.77	6.85
S20	21.4	21.8	21.6	0.12	2.90	-24.75	24.52
S22	22.7	22.9	22.8	0.07	0.11	-25.47	1.48
S23	23.3	23.5	23.4	0.15	0.13	-25.30	0.87
S24	24.2	24.5	24.35	0.05	0.21	-24.86	4.14
S25	24.8	25	24.9	0.02	0.07	-26.48	4.66
S26	25.2	25.5	25.35	0.06	0.46	-25.34	7.94
S27	26	26.3	26.15	0.14	0.22	-25.58	1.60
S28	26.6	26.8	26.7	0.04	0.11	-26.19	2.50
S31	29	29.3	29.15	0.19	0.94	-25.59	4.92
S32	29.5	29.8	29.65	0.14	3.86	-25.67	27.78
S33	30.3	30.5	30.4	0.05	1.01	-26.17	18.92
S34	30.8	31	30.9	0.07	0.73	-25.39	11.24
S39	34.2	34.5	34.35	0.06	1.00	-25.82	16.13
S41	35.2	35.5	35.35	0.03	0.11	-25.69	3.98
S43	36.3	36.6	36.45	0.02	0.10	-26.22	4.97
S44	36.9	37.1	37	0.04	1.05	-25.48	24.52
S45	37.6	37.7	37.65	0.04	0.66	-25.61	18.74
S46	38.3	38.7	38.5	0.15	0.59	-25.97	3.97
S47	38.9	39.2	39.05	0.03	0.41	-26.05	15.86
S48b	40	40.4	40.2	0.02	0.23	-26.12	9.64
S50	41.6	41.9	41.75	0.02	0.10	-25.94	4.49
S51	42	42.2	42.1	0.02	0.16	-25.85	8.47
S52	42.4	42.6	42.5	0.04	0.59	-25.30	14.82
S53	43	43.5	43.25	0.08	0.52	-25.61	6.75
S54	43.8	43.9	43.85	0.03	0.22	-25.98	7.65

S56	44.7	44.9	44.8	0.02	0.23	-25.88	9.45
S58a	45.6	45.9	45.75	0.03	0.67	-25.37	24.49
S58b	46	46.1	46.05	0.02	0.07	-26.52	3.88
S59	46.5	46.7	46.6	0.05	0.62	-25.56	13.76
S60	47	47.1	47.05	0.05	0.20	-26.13	4.09
S61	47.5	47.7	47.6	0.02	0.07	-26.12	3.08
S63	48.5	48.7	48.6	0.02	0.12	-25.43	5.47
S65	49.7	49.9	49.8	0.03	0.59	-25.13	17.90
S66	50.2	50.4	50.3	0.02	0.27	-26.49	11.16
S67	50.7	50.8	50.75	0.02	0.09	-26.91	3.74
S68	51.1	51.3	51.2	0.18	0.11	-26.76	0.61
S69	51.6	51.8	51.7	0.02	0.05	-27.02	2.87
S71	52.8	53	52.9	0.03	0.08	-26.54	2.85
S73	53.8	54.1	53.95	0.04	0.07	-27.36	1.80
S74	54.3	54.5	54.4	0.02	0.03	-27.01	1.79
S75	54.8	55.1	54.95	0.06	0.05	-26.37	0.96
S76	55.2	55.4	55.3	0.05	0.10	-26.06	2.17
S77	55.8	56	55.9	0.02	0.08	-26.29	3.68
S79	56.8	57	56.9	0.02	0.05	-25.60	2.42
S81	57.8	58	57.9	0.06	1.31	-24.69	22.99
S82	58.2	58.4	58.3	0.03	0.02	-27.39	0.78
S83	59	59.2	59.1	0.04	0.69	-25.18	19.12
S84	59.5	59.7	59.6	0.06	0.12	-26.05	2.07
S85	59.9	60.2	60.05	0.02	0.05	-26.54	2.95
S87	61	61.2	61.1	0.02	0.07	-25.89	3.20
S89	61.8	62	61.9	0.02	0.10	-26.39	4.75
S91	62.8	63	62.9	0.02	0.18	-25.72	7.55
S92	63.3	63.5	63.4	0.04	0.10	-26.15	2.39
S93	63.8	64	63.9	0.02	0.11	-26.68	4.95
S95	64.8	65	64.9	0.03	0.07	-26.37	2.62
S97	65.9	66.1	66	0.02	0.07	-25.77	3.28
S98	66.4	66.6	66.5	0.01	0.02	-27.35	1.55
S99	66.9	67.1	67	0.02	0.07	-27.04	4.24
S100	67.3	67.5	67.4	0.03	0.09	-26.07	3.28
S101	67.8	68	67.9	0.02	0.08	-26.78	4.23
S103	69.8	69.2	69.5	0.02	0.08	-26.47	3.44
S105	69.9	70.2	70.05	0.03	0.53	-25.09	15.62
S106	70.4	70.6	70.5	0.02	0.02	-26.64	0.95
S107	71	71.2	71.1	0.02	0.08	-25.44	3.65
S108	71.6	71.8	71.7	0.03	0.15	-25.59	4.83

S109	72	72.2	72.1	0.02	0.06	-26.53	3.23
S110b	72.75	72.85	72.8	0.02	0.07	-26.51	3.27
S111	73.5	73.7	73.6	0.02	0.07	-26.95	3.29
S112	73.9	74.1	74	0.02	0.04	-25.63	2.45
S113	74.35	74.55	74.45	0.02	0.06	-26.59	2.83
S114	74.9	75.1	75	0.04	0.16	-25.45	4.33
S115a	75.4	75.6	75.5	0.02	0.07	-26.72	3.42
S115c	76.1	76.3	76.2	0.02	0.09	-26.04	3.50
S117	76.9	77.1	77	0.02	0.16	-25.94	6.45
S118	77.4	77.6	77.5	0.02	0.02	-26.06	1.40
S119	77.9	78.1	78	0.02	0.08	-27.29	4.23
S120	78.4	78.6	78.5	0.03	0.09	-25.81	2.81
S121	79	79.2	79.1	0.02	0.06	-26.52	3.23
S123	79.9	80.1	80	0.02	0.07	-26.41	4.09
S125	81	81.2	81.1	0.02	0.06	-26.31	3.41
S126a	81.45	81.55	81.5	0.02	0.03	-26.69	2.10
S126b	81.9	82	81.95	0.02	0.08	-25.89	4.47
S127	82.3	82.5	82.4	0.03	0.12	-24.69	3.78
S128	82.9	83.1	83	0.01	0.05	-26.47	3.55
S130	83.9	84.2	84.05	0.04	0.13	-25.93	3.50
S131b	85	85.1	85.05	0.02	0.10	-26.46	4.28
S132a	85.5	85.6	85.55	0.02	0.04	-26.50	2.29
S132b	86	86.2	86.1	0.02	0.10	-25.79	4.49
S133	86.3	86.5	86.4	0.03	0.13	-26.59	4.21
S135a	87.5	87.7	87.6	0.06	0.31	-25.44	4.86
S136	88.4	88.6	88.5	0.05	0.23	-25.14	4.88
S140	90.5	90.7	90.6	0.17	0.61	-24.79	3.67
S141	91.05	91.25	91.15	0.03	0.13	-23.70	4.15
S142	91.6	91.8	91.7	0.04	0.15	-25.26	3.73
S144a	92.5	92.6	92.55	0.02	0.07	-25.53	3.37
S145	93.4	93.6	93.5	0.02	0.07	-25.43	2.99
S146	93.8	94	93.9	0.01	0.03	-25.44	2.23
S147	94.35	94.55	94.45	0.04	0.85	-24.51	22.64
S148	94.8	95	94.9	0.04	0.18	-25.39	4.88
S149	95.3	95.5	95.4	0.03	0.15	-25.22	5.78
S151a	96.5	96.6	96.55	0.02	0.08	-26.04	4.21
S152	97.3	97.5	97.4	0.01	0.07	-26.74	5.02
S153a	97.65	97.85	97.75	0.01	0.02	-25.91	2.49
S153b	98.1	98.2	98.15	0.13	0.07	-25.47	0.57
S154a	98.3	98.4	98.35	0.27	0.95	-23.69	3.50

S155a	98.6	98.7	98.65	0.22	0.67	-25.18	3.11
S156	99.3	99.5	99.4	0.09	0.10	-25.72	1.05
S158	100.4	100.6	100.5	0.07	0.06	-26.18	0.86
S159	101	101.2	101.1	0.03	0.36	-24.21	14.09
S160	101.6	101.8	101.7	0.04	0.05	-26.07	1.24
S161	102	102.2	102.1	0.06	0.21	-24.87	3.27
S162	102.5	102.7	102.6	0.06	0.36	-25.17	6.36
S164	103.5	103.7	103.6	0.02	0.16	-25.59	6.41
S166	104.4	104.5	104.45	0.04	0.07	-25.97	1.92
S167	104.9	105	104.95	0.01	0.02	-25.86	1.49
S168	105.4	105.5	105.45	0.04	0.51	-25.22	12.19
S169	105.9	106.1	106	0.22	0.14	-24.95	0.63
S172	107.5	107.7	107.6	0.03	0.25	-25.77	7.70
S174	108.7	108.9	108.8	0.03	0.12	-26.22	4.74
S175	109.1	109.2	109.15	0.03	0.35	-25.51	14.12
S176	109.5	109.7	109.6	0.04	0.26	-25.65	7.32
S177	109.95	110.05	110	0.32	4.19	-25.20	13.23
S178	110.55	110.65	110.6	0.05	0.82	-25.54	15.85
S180	111.5	111.7	111.6	0.04	0.87	-25.53	19.29
S182	112.8	113	112.9	0.04	0.65	-25.36	17.32
S183	113.6	113.6	113.6	0.04	0.69	-25.72	16.53
S184	114.1	114.1	114.1	0.04	0.56	-25.49	14.43
S185	114.7	114.8	114.75	0.21	3.51	-24.46	17.04
S186	115.2	115.3	115.25	0.03	0.30	-25.64	9.58
S189	116.8	116.9	116.85	0.14	6.67	-25.70	46.02
S192	118.2	118.4	118.3	0.03	0.49	-24.34	14.46
S193	118.75	118.85	118.8	0.04	0.37	-26.18	9.28
S194	119.15	119.25	119.2	0.14	0.35	-24.28	2.50
S195	119.7	119.8	119.75	0.08	2.07	-26.07	26.19
S197	120.6	120.7	120.65	0.08	3.42	-25.70	43.84
S199	121.75	121.85	121.8	0.06	0.63	-26.22	10.92
S200	122.2	122.3	122.25	0.06	0.41	-25.57	7.10
S201	122.6	122.7	122.65	0.04	0.28	-25.93	6.49
S202	122.9	123	122.95	0.13	0.87	-24.74	6.68
S203	123.3	123.4	123.35	0.02	0.09	-26.41	4.27
S205	124.1	124.2	124.15	0.02	0.09	-26.85	5.09
S207	125.3	125.4	125.35	0.02	0.08	-26.55	4.00
S208	125.7	125.8	125.75	0.02	0.02	-26.60	0.92
S209	126.2	126.3	126.25	0.02	0.04	-26.08	2.06
S210	126.7	126.8	126.75	0.10	0.28	-24.13	2.68

S211	127.2	127.4	127.3	0.02	0.12	-26.13	7.78
S213	128.1	128.2	128.15	0.02	0.05	-26.08	2.73
S215	129.1	129.4	129.25	0.05	3.21	-25.40	61.67
S216	129.4	129.5	129.45	0.03	1.73	-25.35	64.33
S217	129.7	129.9	129.8	0.02	0.08	-26.64	4.23
S218	130.2	130.4	130.3	0.10	0.12	-24.88	1.21
S219	130.6	130.7	130.65	0.02	0.05	-25.76	3.04
S221	131.7	131.8	131.75	0.03	0.61	-25.87	21.08
S223	132.9	133.1	133	0.02	0.05	-26.53	2.90
S225	133.8	134	133.9	0.02	0.05	-26.21	2.31
S226	134.4	134.5	134.45	0.11	0.12	-25.49	1.08
S227	134.8	134.9	134.85	0.05	0.80	-25.11	14.88
S229	135.8	135.9	135.85	0.07	0.89	-25.26	12.32
S231	136.7	136.8	136.75	0.04	0.91	-25.63	22.44
S232	137.15	137.25	137.2	0.05	0.61	-25.20	13.45
S233	137.65	137.75	137.7	0.04	0.46	-25.61	10.46
S234	138	138.1	138.05	0.11	0.43	-24.62	3.96
S235	138.4	138.5	138.45	0.04	0.49	-25.67	13.42
S237	139.2	139.3	139.25	0.04	0.56	-25.69	13.08
S239	139.75	139.85	139.8	0.04	0.41	-25.74	11.56
S240	140.3	140.4	140.35	0.05	0.26	-25.88	5.43
S241	140.65	140.85	140.75	0.04	0.67	-25.65	16.05
S242	141.2	141.4	141.3	0.16	0.35	-24.06	2.21
S243	141.75	141.85	141.8	0.02	0.06	-27.32	2.69
S245	142.8	143	142.9	0.02	0.07	-27.20	3.54
S247	143.9	144	143.95	0.02	0.08	-27.10	3.63
S248	144.4	144.5	144.45	0.02	0.04	-26.47	2.15
S249	144.8	144.9	144.85	0.02	0.06	-26.66	3.18
S250	145.3	145.3	145.3	0.03	0.18	-25.46	5.78
S251	145.9	146	145.95	0.02	0.05	-27.15	2.43
S253	146.85	146.95	146.9	0.02	0.09	-27.13	4.08
S255	148.2	148.4	148.3	0.02	0.17	-26.39	10.15
S256	148.85	148.95	148.9	0.05	1.01	-25.88	18.54
S257	149.05	149.15	149.1	0.01	0.07	-27.11	4.93
S258	149.4	149.6	149.5	0.02	0.08	-26.31	3.37
S259	149.8	150	149.9	0.04	0.59	-25.60	16.19
S261	150.6	150.7	150.65	0.02	0.06	-26.44	2.82
S263	151.7	151.8	151.75	0.03	0.22	-25.90	7.88
S264	152.1	152.2	152.15	0.03	0.16	-26.27	6.20
S265	152.6	152.7	152.65	0.03	0.12	-26.51	4.13

S266	153.05	153.15	153.1	0.14	0.14	-26.59	0.97
S267	153.65	153.75	153.7	0.02	0.06	-26.76	3.95
S269	154.6	154.8	154.7	0.10	4.54	-25.17	44.42
S271	155.7	155.7	155.7	0.13	6.27	-25.09	47.36
S272	156.1	156.2	156.15	0.02	0.04	-27.29	1.99
S273	156.4	156.5	156.45	0.02	0.09	-27.05	4.10
S274	157	157.1	157.05	0.12	0.17	-28.51	1.41
S275	157.5	157.6	157.55	0.02	0.08	-27.09	3.34
S277	158.42	158.52	158.47	0.02	0.07	-26.83	3.14
S279	159.4	159.5	159.45	0.02	0.09	-26.52	4.35
S280	160	160.1	160.05	0.02	0.04	-27.04	2.18
S281	160.7	160.8	160.75	0.02	0.09	-26.43	4.20
S282	161	161.1	161.05	0.03	0.15	-27.03	4.48
S283	161.4	161.5	161.45	0.02	0.11	-26.73	5.40
S285	162.4	162.5	162.45	0.02	0.14	-26.15	6.80
S287	163.4	163.5	163.45	0.03	0.21	-25.33	8.07
S288	163.9	164	163.95	0.02	0.03	-27.22	1.60
S289	164.1	164.2	164.15	0.02	0.06	-26.68	3.23
S290	164.5	164.6	164.55	0.04	0.15	-27.59	4.35
S291	164.9	165	164.95	0.02	0.07	-26.85	3.78
S293	166	166.2	166.1	0.02	0.07	-26.63	3.85
S296	167.4	167.5	167.45	0.08	3.62	-26.12	47.64
S297	167.9	168	167.95	0.11	4.78	-25.10	44.99
S298	168.2	168.3	168.25	0.26	8.15	-25.99	31.12
S299	168.55	168.65	168.6	0.05	2.04	-25.42	41.59
S306	172.1	172.2	172.15	0.26	5.92	-25.65	22.65
S312	174.9	175.1	175	0.02	0.12	-26.93	5.36
S314	175.85	175.95	175.9	0.18	0.18	-24.24	1.01
S320	179.3	179.5	179.4	0.03	0.19	-25.76	6.81
S322	180.5	180.7	180.6	0.13	0.12	-25.40	0.90
S329	183.9	184.1	184	0.07	0.91	-24.87	13.91
S331	185	185.1	185.05	0.05	0.38	-25.32	7.85
S337	187.5	187.7	187.6	0.13	7.77	-25.75	58.51
S356	198.2	198.3	198.25	0.02	0.13	-26.36	6.02
S358	199.1	199.2	199.15	0.07	0.61	-26.14	9.15
S362	201.05	201.15	201.1	0.04	7.29	-25.51	180.84
S364	202.05	202.15	202.1	0.05	0.42	-26.00	8.83
S366	202.9	203	202.95	0.08	0.55	-26.82	7.20
S372	206.1	206.3	206.2	0.04	0.46	-26.58	10.87
S374	207.1	207.2	207.15	0.08	0.59	-26.15	7.78

S382	209.9	210.1	210	0.12	5.36	-26.46	43.75
S390	213.5	213.7	213.6	0.01	0.20	-26.26	16.61
S397	217.5	217.6	217.55	0.04	0.12	-25.24	2.89
S399	218.4	218.5	218.45	0.06	0.69	-27.44	11.62
S405	221.3	221.4	221.35	0.04	0.81	-25.75	18.31
S407	222.4	222.5	222.45	0.05	0.57	-25.79	11.72
S413	225.15	225.35	225.25	0.02	0.40	-27.38	17.17
S415	226.3	226.4	226.35	0.05	0.51	-27.07	10.36
S421	229.3	229.4	229.35	0.03	0.44	-27.21	14.06
S423	230.3	230.4	230.35	0.05	0.40	-25.08	7.77
S431	234.3	234.4	234.35	0.15	0.51	-25.77	3.49
S437	237.3	237.4	237.35	0.04	0.39	-25.08	11.18
S439	238.1	238.2	238.15	0.05	0.29	-24.42	5.95
S445	240.6	240.8	240.7	0.03	0.42	-25.24	12.80
S447	241.5	241.7	241.6	0.07	0.50	-25.66	7.44
S453	244.3	244.4	244.35	0.02	0.41	-26.31	21.47
S455	245.4	245.5	245.45	0.08	0.77	-27.03	9.45
S461	249.1	249.3	249.2	0.08	1.02	-26.30	12.70
S463	250.95	251.05	251	0.19	0.75	-27.01	4.05
S469	253.8	253.9	253.85	0.06	0.88	-26.36	13.67
S471	254.8	255	254.9	0.04	0.47	-26.95	11.05
S477	257.8	257.9	257.85	0.09	1.24	-27.33	14.10
S479	258.8	259	258.9	0.04	0.49	-27.30	13.24
S485	261.8	262	261.9	0.03	0.14	-25.82	4.26
S487	262.95	263.05	263	0.07	0.45	-26.74	6.56
S493	264.7	264.9	264.8	0.05	0.52	-23.02	9.66
S495	265.7	265.8	265.75	0.03	0.10	-27.15	3.31
S502	269.4	269.5	269.45	0.02	0.16	-24.35	10.12
S504	270.2	270.3	270.25	0.03	0.36	-26.48	11.83
S510	273.3	273.4	273.35	0.04	0.55	-24.65	12.61
S512	274.1	274.3	274.2	0.05	0.41	-27.02	9.14
S518	277.3	277.5	277.4	0.04	0.54	-24.80	14.88
S520	278.2	278.3	278.25	0.13	0.38	-25.06	3.02
S526	281.2	281.3	281.25	0.03	0.34	-25.16	10.08
S528	282.1	282.3	282.2	0.06	0.42	-24.58	7.60
S542	289.1	289.25	289.175	0.02	0.81	-25.45	32.49
S550	293.2	293.3	293.25	0.04	0.04	-26.82	0.97
S552	294.2	294.3	294.25	0.04	0.25	-27.39	6.75
S558	297.2	297.3	297.25	0.01	0.36	-25.24	35.71
S560	298.2	298.3	298.25	0.04	0.46	-26.94	10.45

Correlation coefficient (r) of $\delta^{13}C_{TOC}$ and C/N = 0.19

Coefficient of determination (r^2) of $\delta^{13}C_{TOC}$ and C/N = 0.038
