# Supplementary material for: Carbon cycle perturbations and environmental change of the middle permian and Late Triassic Paleo-Antarctic circle
Source: Sci Rep. 2024 Apr 28;14:9742. doi: 10.1038/s41598-024-60088-5 (PMC11056376; doi:10.1038/s41598-024-60088-5)
Supplement: Supplementary file 2 — Supplementary Information 2. [file 41598_2024_60088_MOESM2_ESM.docx]

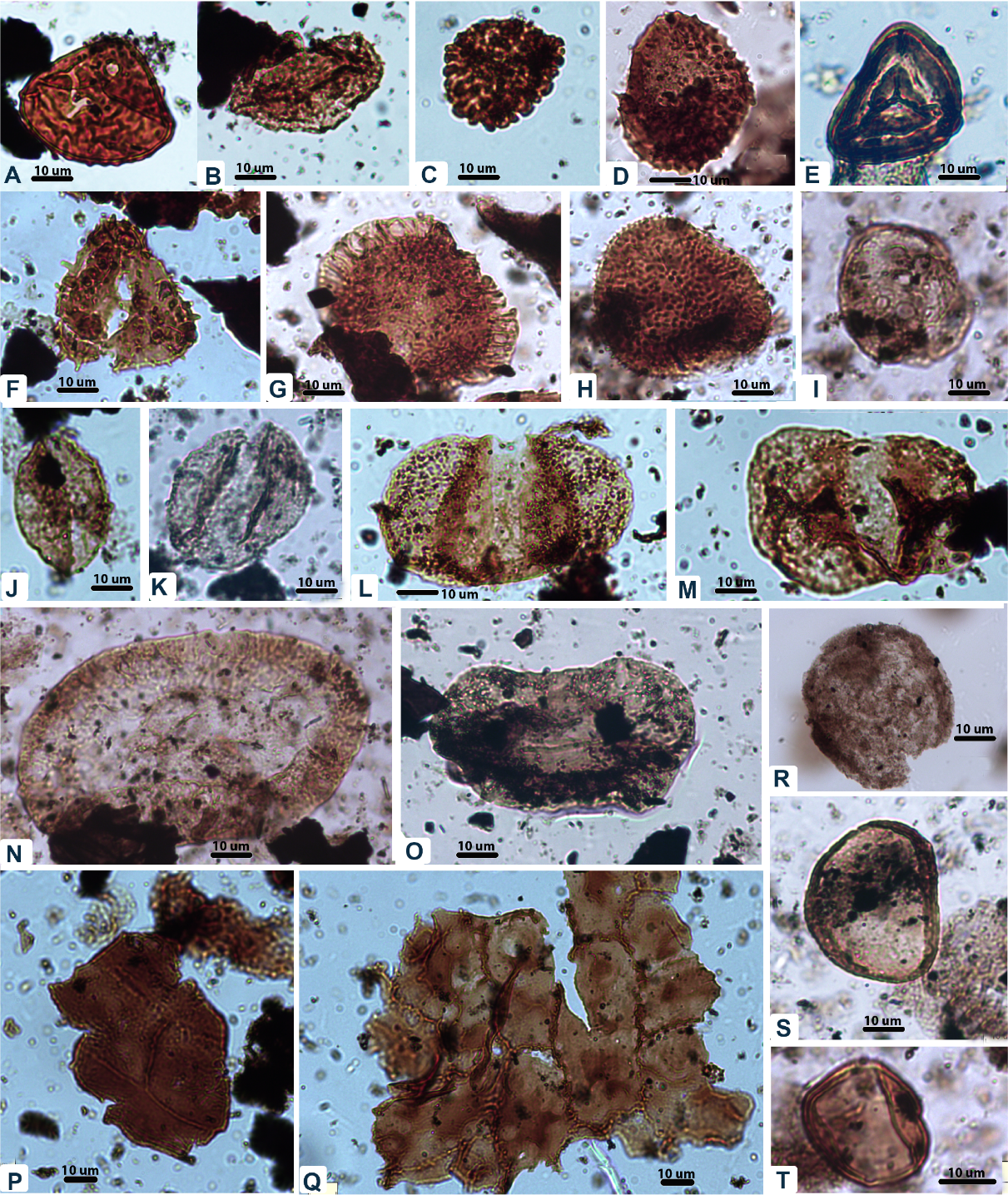


**FIG. SI-1.** The Palynomorphs Plate includes several important taxa. **A**. *Camarozonosporites rudis* (Leschik 1955) Klaus 1960, 83.4 m*;* **B.** *Aratrisporites* *minimus* Schulz 1967*,* 83.4 m; **C.** *Uvaesporites argenteaeformis* (Bolkhovitina 1953) Schulz 1967, 83.6 m; **D.** *Apiculatisporites* sp., 120.15 m; **E.** *Striatella seebergensis* Mädler, 1964; 120.15 m; **F.** *Acanthotriletes microspinosus* (Ibrahim, 1933) Potonié & Kremp, 1955; 200.05 m; **G.** *Limbosporites denmeadii* (de Jersey 1962) de Jersey & Raine 1990, 153.3 m; **H**. *Osmundacidites wellmanii* Couper 1953, 154.1 m; **I.** *Craterisporites rotundus* de Jersey, 1970, 167.4 m; **J.** *Monosulcites* spp**.**, 83.4 m; **K.** *Scheuringipollenites ovatus*, 233.3 m; **L.** *Alisporites australis* de Jersey 1962, 120.15 m; **M.** *Pteruchipollenites* sp., 154.1; **N.**  *Parasaccites* spp. 233.3 m; **O**. *Staurosaccites quadrifidus,* 167.4*;* **P.** cuticle of *Dicroidium* sp. **Q.** cuticle of *Dicroidium odontopteroides*Morris 1845, 83.4; **R.** *Tasmanites* sp*.,* 289.1 m; **S-T.** *Pilasporites calculus* Balme and Hennelly 1956; 233.3 m.


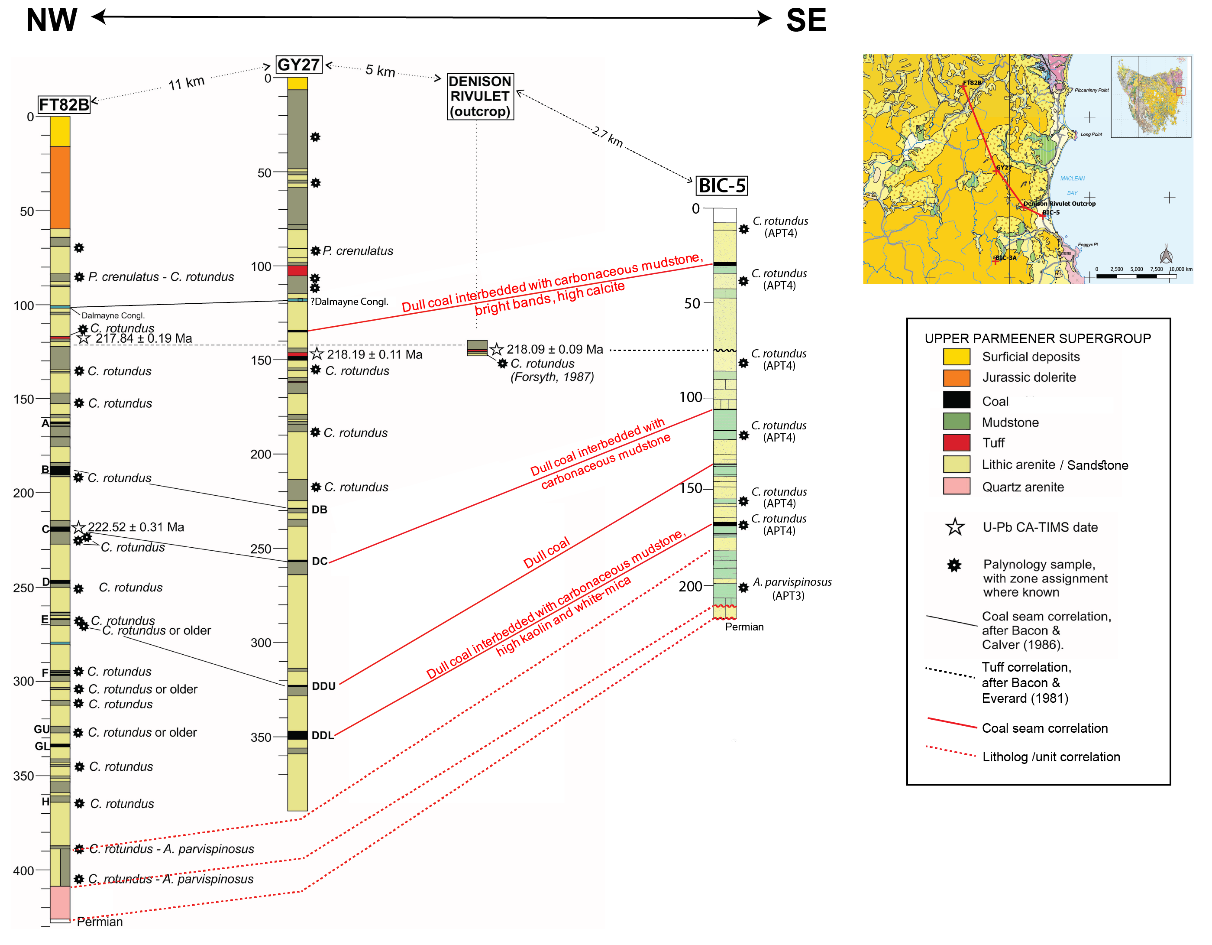


**FIG. SI-2** Supplementary figure showing the location of core sites (inset adapted from 1:250,000 Digital Geological Data from from www.mrt.tas.gov.au, State of Tasmania). Detailed lithostratigraphy and chemostratigraphy correlation, including several coal seams, were performed between the Bicheno-5 (BIC-5) and GY27 detailed borehole description after Ford and Bos (1985). Palynological results Bicheno-5 support the correlation with nearby deposits of FT82B, GY27, and Denison Rivulet outcrops (after Calver et al., 2021). Detailed Bicheno-5 lithology can be found in Fig. 3 and Fig 4.


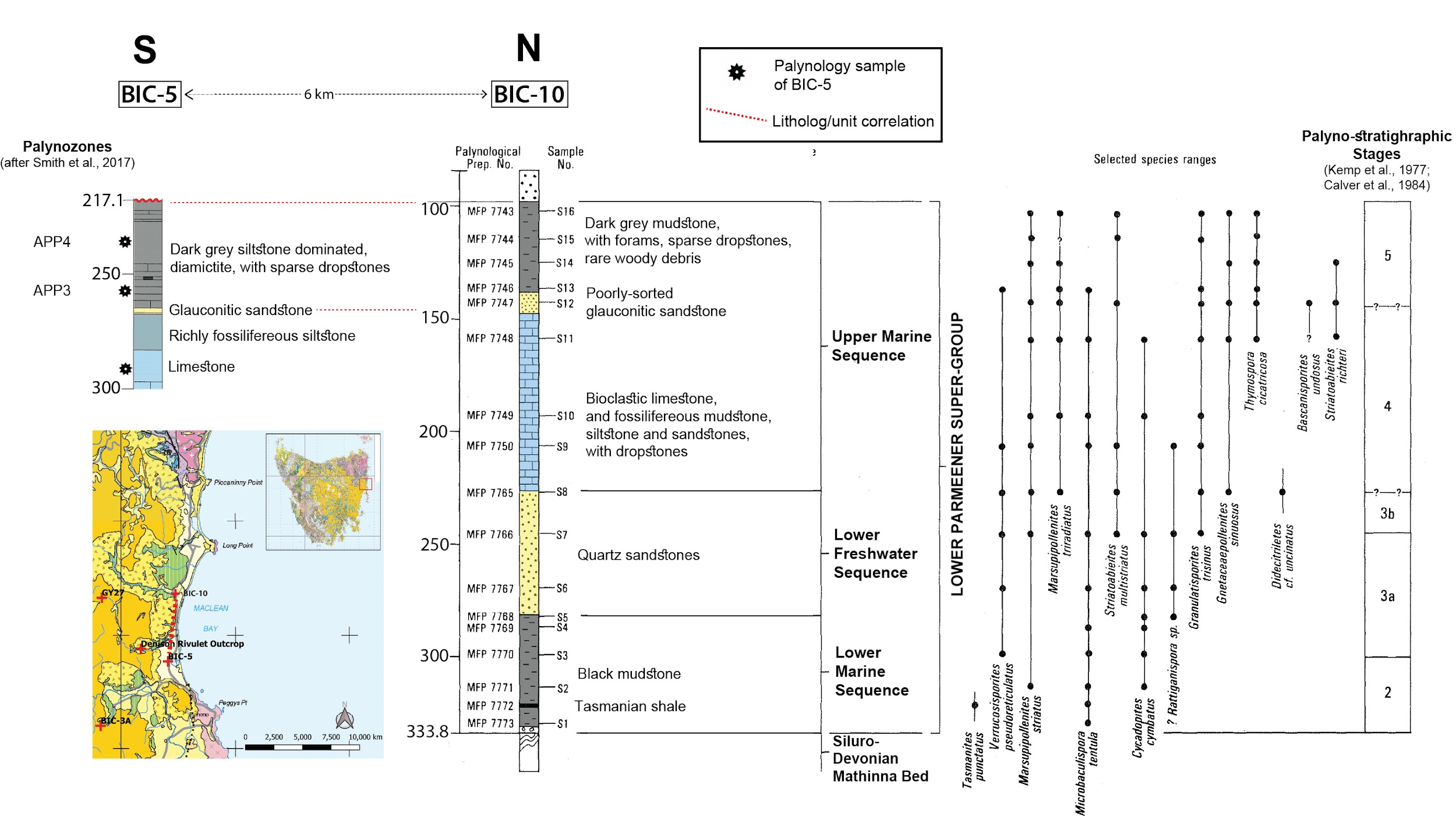

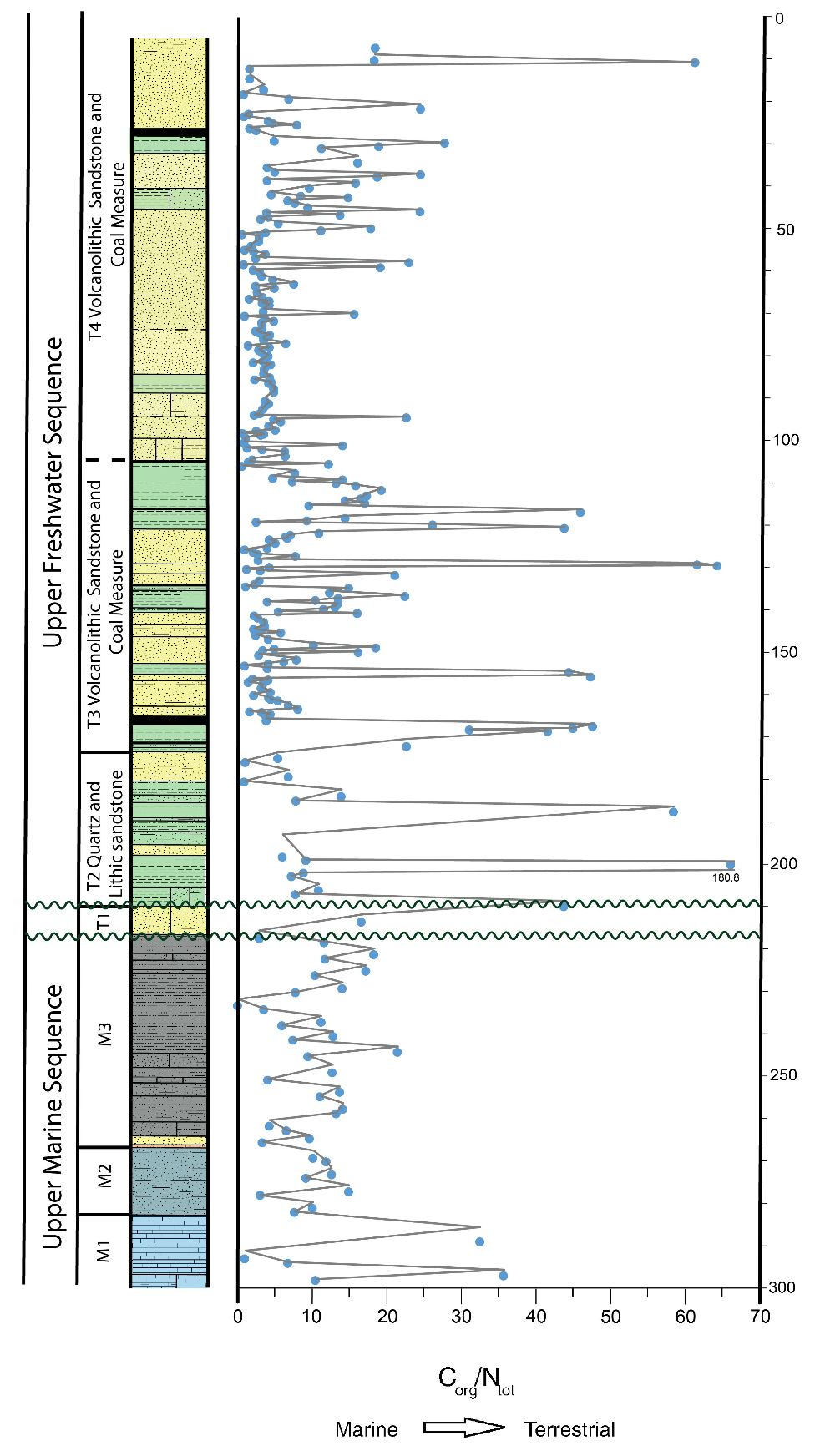


**FIG. SI-3.** Supplementary figure showing the location of core sites (inset adapted from 1:250,000 Digital Geological Data from from www.mrt.tas.gov.au, State of Tasmania). Bicheno-10 (BIC-10) based on palynostratigraphic stages boundaries on the right-hand side (after Kemp et al., 1977; Calver et al., 1984), which coherence with the palynology study in Bicheno-5 (BIC-5) showing APP 4 and APP 3 (left-hand side, data is from this study ) from The Australian Guadalupian to Lopingian, Permian palynostratigraphic zonation (after Smith and Mantle 2013; Smith et al.,2017).

**FIG. SI-4** The graph displays C_org_/N_tot_ ratios at different depths in the Bicheno-5 section. The typical C_org_/N_tot_ ratio of marine and terrestrial organic matter follows Meyers (1997).


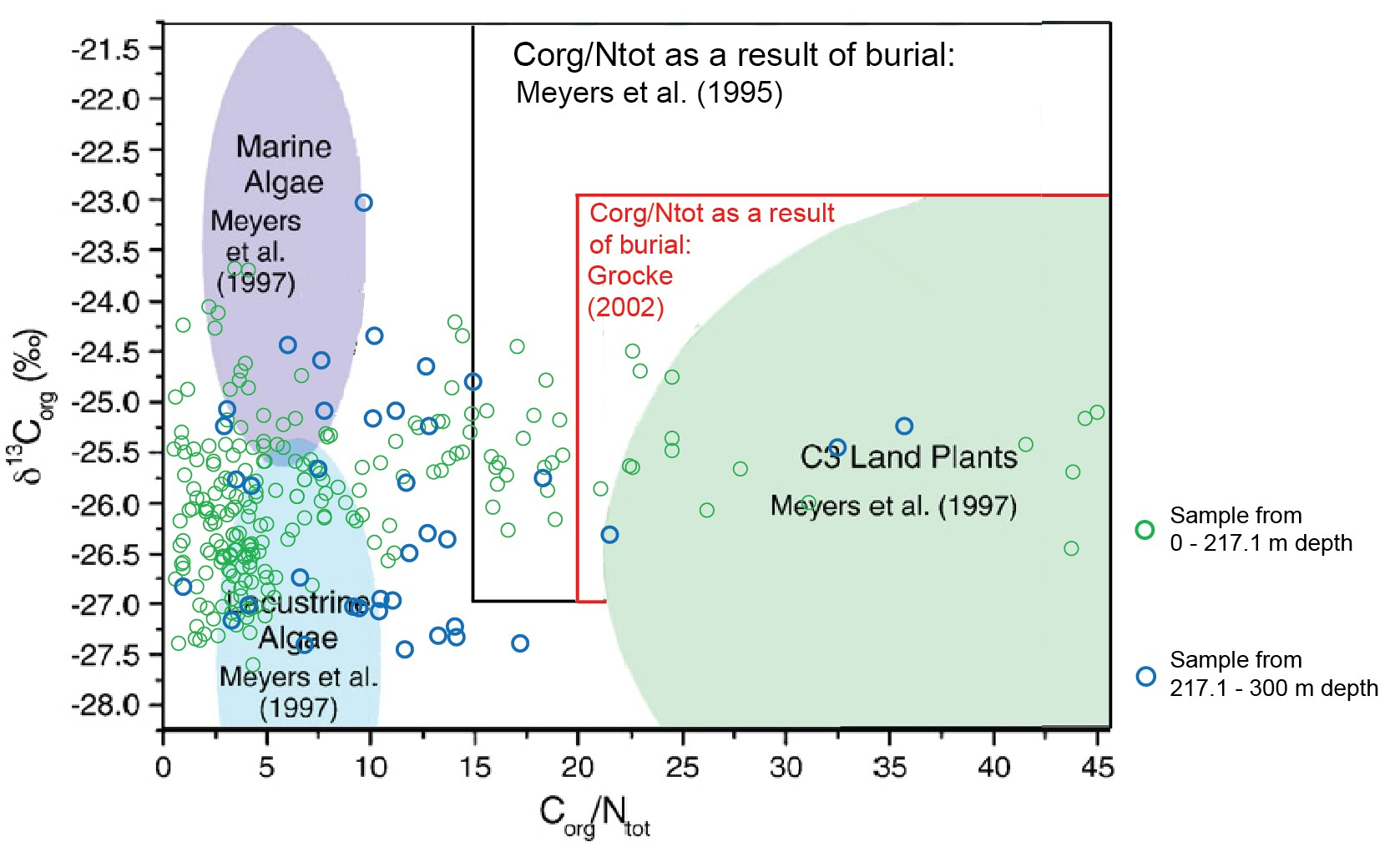


**FIG. SI-5** Cross plots of total organic carbon content (% C), total nitrogen content (% N_tot_), C_org_/N_tot_, and δ13Corg data obtained from the Bicheno-5 section. Samples from a depth of 0 to 217.7m (Triassic) are green open circles, and samples from 217.1 to 300m (Middle Permian) are blue open circles. Plotted fields are based on Meyers (1997) and Gröcke (2002).
